# The Fold-Illuminator: A low-cost, portable, and disposable incubator-illuminator device

**DOI:** 10.1016/j.synbio.2021.04.003

**Published:** 2021-04-23

**Authors:** Logan R. Burrington, Emran Baryal, Katherine Hui, Emmett Lambert, Sarah T. Harding, Javin P. Oza

**Affiliations:** a Chemistry and Biochemistry Department, College of Science and Math, California Polytechnic State University, San Luis Obispo, CA, 93407, USA; b Mechanical Engineering Department, College of Engineering, California Polytechnic State University, San Luis Obispo, CA, 93407, USA

**Keywords:** Illuminator, Incubator, Fluorescent reporter, Education, Diagnostics, Frugal science

## Abstract

Fluorescent reporters have revolutionized modern applications in the fields of molecular and synthetic biology, enabling applications ranging from education to point-of-care diagnostics. Past advancements in these fields have primarily focused on improving reaction conditions, the development of new applications, and the broad dissemination of these technologies. However, field and classroom-based applications have remained limited in part due to the nature of fluorescent signal detection, which often requires the use of costly lab equipment to observe and quantify fluorescence readouts. Users without access to laboratory equipment rely on qualitative assessments of fluorescence, a process that remains highly variable from user-to-user even within the same classroom. To overcome this challenge, we have developed a foldable illuminator and incubator device to support field-applications of synthetic biology-based biosensors for education and diagnostics. The Fold-Illuminator is an affordable, portable, and recyclable device that allows for the visible detection of fluorescent biomolecules. The Fold-Illuminator's design allows for assembly in under 10 min, a user can then utilize the optional heating element to incubate biochemical reactions and visualize fluorescence outputs in a defined and light-controlled environment. Interchangeable LED strips and light-filtering screens provide modularity to pair with the fluorescence wavelengths of interest. The user can then unfold the device for convenient storage, transport, or even recycling. The cost for the Fold-Illuminator is $5.58 USD and is compatible with an optional heating element for an additional $3.98 cost, with potential for further reductions in cost for larger quantities. Open-source templates for cutting device parts from paper stock are provided for both printing and cutting by hand; cutting can also be achieved with consumer-grade smart cutting machines such as the Cricut®. Combined with the broad applications of fluorescent reporters, the Fold-Illuminator has the potential to improve access to fluorescence visualization and quantification for new users as well as emerging field applications.

## 1 Introduction

Fluorescent reporters have transformed the way that many biochemical processes are applied in the real-world. In recent years, fluorescent reporters have enabled many novel applications in diagnostics [[1], [2], [3]], point-of-care testing [4] and education [5]. The development of reporters, which range in composition and mechanism from small molecule fluorophores and fluorogens to nanomaterials and fluorescent proteins [6], has allowed for the observation of biochemical processes in real time [7]. While the fluorescent reporter and the application may vary, each of these cases represent a dependency on laboratory equipment for the reproducible visualization and quantification of fluorescence signals.

We sought to develop a low-cost portable fluorescence illuminator that could provide a consistent environment for the incubation and visualization of biochemical reactions for field-based applications. Specifically, we sought to reduce the most common sources of variability in field-observations of fluorescence in the absence of instrumentation – variability in excitation light source, angle, and intensity, as well as the variability in ambient light. Notably, portable illuminators have been previously developed (Table S1) [8], [9], [33], [34]. However, we have pursued a solution to the aforementioned objectives through an alternate design approach that increases compatibility with classrooms and low-resource communities.

Capital cost of equipment is only one aspect of the limitations facing implementation in low-resource environments. For example, over 90% of medical equipment donated to low- and lower-middle-income countries (LMICs) is projected to be non-functional within five years [10]. Reliability and low-cost are important features in a portable device, but insufficient for broader impacts. Design must anticipate that 1) malfunctions must not present catastrophic damage to the device or the user, 2) replacement parts can be easily sourced, and 3) repairs can be made with common technical skills and resources. A notable example includes the NeoNurture, a neonatal incubator made from car parts such that repairs can be made by a local workforce skilled in automotive repair using available car supplies [11]. Similar principles apply for the democratization of laboratory equipment. Low-cost open-source labware is essential for field-applications as well as implementation of emerging science in the LMICs and global education [12,13]. Toward this end, there is interest in paper-based, foldable devices that provide the utility of traditional labware, with the advantages of portability, cost, and materials availability that support broad dissemination [[14], [15], [16], [17]].

To develop a low-cost, portable fluorescent illuminator, it's important to distinguish between quantitative and qualitative fluorescence detection. Quantitative methods report exact concentrations of fluorescent reporters but are often costly and lack portability [[18], [19], [20]], whereas qualitative methods are generally cheap and portable [21,22]. Qualitative detection often relies on the use of eyesight, where the user is able to confirm a fluorescence signal in the visible spectrum, and can be augmented with the use of a light source matching the excitation wavelength and a filter to improve the signal-to-noise ratio compared to ambient light alone. The resulting improvements to the sensitivity of visible observations means that the user can score for success in significantly less time compared to ambient light conditions. Costing on average less than $3.00 for the pair, our design constraints utilize a LED/light filter combination as an affordable, portable option that would allow the user to obtain more confidence in their reaction results in less time. Additionally, biochemical reactions such as cell-free protein synthesis benefit from incubation at temperatures in the range of 30–37 **°**C [4], [5]. While not all applications may require a built-in incubator, our design constraints required compatibility with this optional add-on.

Further features that characterize the Fold-Illuminator are durability, affordability, and recyclability. After numerous iterations, we report a design that is user-friendly, meets our design criteria, and costs $5.58 per Fold-Illuminator, with an additional $3.98 for the optional USB-powered heating element. The entire exterior is paper based, offering 85% recyclability by weight and a durable design to prevent catastrophic loss of function upon dropping. Considering that most of the design is paper-based, new parts can be easily constructed from available paper and cardstock. Combined, these features allow for a device that can be disseminated into the hands of users globally. Here we detail the design, production, and assembly of the Fold-Illuminator. We include user manuals and videos for instructors and technical staff, as well as for the users themselves.

### 1.1 Current transilluminators, diagnostic devices, and portability innovations

Over the past several years, advancements in molecular biology techniques and the increasing portability of these techniques have ushered the science out of the laboratory and into the field. The adaptation of techniques like microscopy and cell-free expression as diagnostics assays that traditionally required laboratory-grade equipment and trained technical staff into hand-held, user-friendly devices has driven the need for lower cost, portable devices ideal for classroom and field-based use [14]. Naturally, the demand for access to cheap, portable, equipment has also increased as new users have become interested in the educational and life-saving benefits of these enabling technologies.

The portability offered by the Fold-Illuminator reported here was inspired through past advancements in the conversion of laboratory-grade equipment into portable equipment accessible to field and classroom-based users [[14], [15], [16]]. The Foldscope, for example, is a paper origami-based microscope that has significantly increased the access to microscopy around the world for science and education [14]. Starting from a flat piece of paper, assembly time takes less than 10 min while allowing for up to 2000× magnification. Inexpensive microscopes that cost ~$15 are readily available, while the Foldscope reduces that cost by 10x. In addition to cost-effectiveness, the portability, and construction from readily available materials has allowed for the Foldscope's broad dissemination and its corresponding impact as an educational tool as well as a low-cost diagnostic tool [23]. Following the success of the Foldscope, the Paperfuge provides the capacity to perform centrifugation of biological samples in the field and classrooms at a low cost, and with the ease-of-use equivalent to a children's toy [17]. While these specific examples were a source of our inspiration, similar design thinking has been applied towards the development of a variety of devices, even for complex devices such as foldable, functional robots [15,16].

Fluorescence detectors have also been previously integrated into portable systems. A recently developed pocket-sized fluorescence detector was implemented as an affordable method to detect fluorescence in point-of-care testing [24]. The samples are first assayed on filter paper, which is then placed in the device for product detection. Costing less than $15, the device utilizes LEDs, a light dependent resistor, and filter foil for fluorescence detection and can reach a lower limit of detection of 6.8 nM fluorescein. By using a Cas13a-based fluorescence assay, this limit of detection was decreased to 3.7 nM. However, this device is limited to the detection of paper-spotted reactions. Portable devices for visualizing fluorescence from cell-free systems capable of accommodating microfuge tubes and 96-well plates have also been developed, and have demonstrated the utility of a portable illuminator for education and point-of-care applications [9]. Design features of these devices are useful and were also considered in the development of the Fold-Illuminator, as we aimed to transition from their plastic or 3D printed parts to paper-based scaffolds.

Paper has proven to be an important material for synthetic biology more broadly. Many other portable diagnostic and biosensor tools rely on paper as the scaffold for containing and implementing biochemical reactions; while these approaches are distinct from the Fold-Illuminator device reported herein, we believe it is important to note the powerful utility of paper for field applications of biotechnology. In many cases, paper is used to replace the traditional reaction vessels such as microfuge tubes and microfluidic devices. Paper-based reactions often employ lyophilized cell-free expression alongside synthetic diagnostic systems, such as CRISPR-Cas, toehold switch, or riboswitch-based systems [3,25]. Due to their being run on paper, reaction costs are cheap – often offering material costs of less than $10 USD per reaction [1,25]. With applications ranging from detection of water contaminants, nucleic acids, proteins, microbes, and a variety of viruses, these paper-based diagnostics take traditionally laboratory-based experiments and make them deployable to field and classroom-based settings [1,2,14,25,26].

Observing commonalities among existing fluorescence detectors allowed us to establish key design constraints, for example, LEDs coupled with a light-filtering screen appeared to be the most common low-cost method for exciting the fluorophore and observing emission. The Fold-Illuminator is also paper-based, allowing for a cheap, foldable, and portable design ideal for field and classroom users. Lastly, as opposed to other currently available fluorescence illuminators, the Fold-Illuminator has been designed to accommodate a USB-powered heating element for reaction incubation. The culmination of all these features has resulted in a novel design ideal for many applications of molecular and synthetic biology.

## 2 Design and construction

**Fig. 1:**
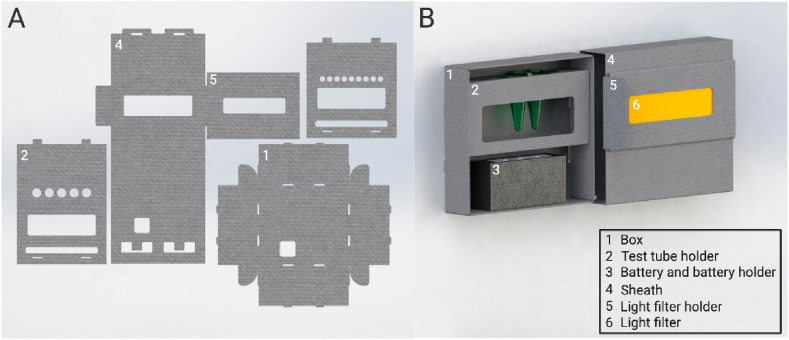
(A) Schematic of the Fold-Illuminator paper-based exterior. (B) Depiction of the complete Fold-Illuminator design, which includes the paper-based box exterior, test tube holder, sheath, light filter holder, light filter, battery, and battery holder without the optional heating element. More detailed designs can be found in Supplementary Material Fold-Illuminator Design.

The components of the Fold-Illuminator (Table 1) can be collected into a kit, which the user can assemble when needed. All listed components are modular, with the LED-strip and acrylic filters commercially being to support the desired fluorescence readout. Instructions on kit set-up are included in both a file (Supplementary Material Fold-Illuminator Assembly Instructions) and video (Supplementary Video 1) format. New users can expect set-up to take under 10 min, with the final product being a 4.5 × 4 × 1-inch easy-to-carry rectangular device weighing ~0.6 pounds (Fig. 1). Detailed designs of individual components can be found in Supplementary Material Fold-Illuminator Design. Templates for cutting device parts from paper stock are provided for both printing and cutting by hand (Supplementary Material: Fold-Illuminator Assembly Instructions); machine cutting can also be achieved using consumer-grade smart cutting machines such as the Cricut® (Supplementary Material The Cricut, Supplementary Video 2). Operational and safety instructions of the Fold-Illuminator can be found in Supplementary Material Operation and Safety. After use, the entire exterior can be disassembled, allowing for easy storage, transport, or recycling.

**Table 1:** Cost of individual Fold-Illuminator components. Shipping and tax is not included. Item descriptions and vendors can be found in Table S2.

Item	Description	Quantity	Cost per unit
Illuminator			
490 nm Blue LED light strip	10 mm width, 3 inch length	1	$1.45
Battery	9 V	1	$1.38
(Amazon Brand)			
Battery Holder	9 V snap connection with switch	1	$0.85
Paperboard	11″ × 24″	1	$0.27
Filter Sheet	1/8″ thick, 4″ × 2″	1	$0.64
Velcro Dots (pair)	0.75″ diameter	2	$0.09
LED to Wire Connector	10 mm LED strip, plastic	1	$0.90
**Summary:**			**Cost per Illuminator**
			$5.58
Item	Description		Cost per unit
**Heating element (optional)**			
USB heater	5 V, carbon fiber, 3.74" × 2.56″	1	$3.98
**Summary for incubator + illuminator**			**Total cost per Illuminator and heater**
			$9.56

Supplementary video related to this article can be found at https://doi.org/10.1016/j.synbio.2021.04.003

The following are the supplementary data related to this article:

Video 1 2 Video 1

Video 2 3 Video 2

### 2.1 Assembly

The origami-type nature of the Fold-Illuminator was made possible through the use of a paper-based exterior design. The Fold-Illuminator exterior comes direct to the user as a kit containing the unassembled box piece, sheath piece, a 0.6 mL microfuge tube holder (9-hole), and a 2.0 mL microfuge tube holder (5-hole) (Fig. 1A). Assembly of these components into the complete Fold-Illuminator design (Fig. 1B) is possible solely through the use of folding, without the need for tape, glue, or any other adhesive material. Obviating the need for adhesives was a design decision intended to improve functionality in resource-limited environments. The battery, LEDs, and or USB heater can then be easily attached to the box piece with the use of provided Velcro dots. Hook and loop tape or a similarly functioning item can act as a Velcro dot replacement; however, double-sided tape is not recommended as a replacement. The acrylic filter simply slides and locks into the light filter holder of the sheath piece. Detailed instructions on assembling the Fold-Illuminator using the kit components are included in Supplementary material 1 and Supplementary Video 1; instructions are also publicly available on GitHub at https://github.com/CPilluminators/Fold-illuminator. Following assembly, the Fold-illuminator is ready for the user to insert their tubes into the tube holder corresponding to their tube size, place it into the box, positioned above the battery, and slide the box into the sheath piece. A hole in the back of the box piece allows the user to then switch on the battery to power the LEDs, enabling visualization of the tubes of interest through the acrylic filter. After use, the Fold-Illuminator can be disassembled following the assembly steps in reverse order, allowing for storage, transport, or recycling of the now compact components.

### 2.2 Cost

The Fold-Illuminator offers a design utilizing affordable materials, costing the user $5.58 per illuminator with an optional $3.98 cost for the heating element (Tables S2 and S3). This price may fluctuate depending on the specific lighting and light-filtering needs of the user, the vendor and shipping costs, and price can be reduced larger quantities of parts are purchased.

## 3 Detection

**Fig. 2:**
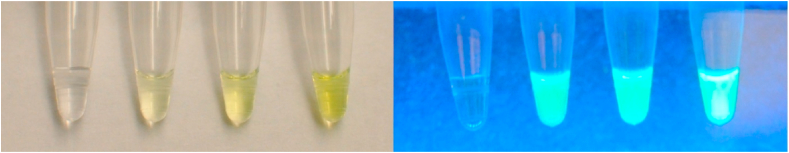
Comparison of sfGFP fluorescence visualization under ambient low-light conditions (left) versus blue light conditions and a yellow acrylic filter using the Fold-Illuminator (right). Both conditions have a negative control on the left, followed respectively by sfGFP concentrations of 125 ng/uL, 250 ng/uL, and 500 ng/uL.

The detection is intended to be qualitative but can become quantitative when combined with image analysis software such as ImageJ. For many applications in education and diagnostics, the goal is often to simply determine whether the reporter is present in the sample, granting the user a yes or no answer to the question: Did my reaction work? This provides less information to the user about the nature of the sample being analyzed, while also minimizing the cost of the analysis. Given that the choice of DNA template and composition of the cell-free system, a yes/no binary readout can provide sufficient, actionable information. For example, a non-expert in a LMIC can evaluate whether their water is safe to drink, or a student can evaluate whether their CRISPR/Cas reaction was successful. The Fold-Illuminator also provides a light-controlled environment for analyzing their samples. The outcome is improved signal-to-noise ratio for viewing fluorescence, which functionally improves the relative sensitivity of the process. (Fig. 2).

### 3.1 LEDs

When a fluorescence reporter is present in robust quantities, ambient white light conditions can be sufficient to visualize the sample (Fig. 2)**.** However, exciting the fluorophore with a dedicated light source can significantly improve the signal output (Fig. 2)**.** Here, LED light strips were chosen to be used in the Fold-Illuminator as an alternate source of light due to their low-cost, compact size, interchangeability, and user-friendliness. The vendor was Waveform lighting, who charged $1.45 per 10 mm width, 3 inch length blue LED light strip (Table S3). As the entire Fold-Illuminator setup is user-defined, changing the LED bulb type only requires ordering a different LED strip. The modularity of this feature lends itself to the effectiveness of the Fold-Illuminator across a plethora of fluorescent reporters and reaction types. In our evaluations, we have found that fewer LEDs suffice for visualizing the sample and may even reduce background noise. Users can optimize the LED strip length for their purposes for both improving signal-to-noise as well as reducing overall cost.

### 3.2 Light filtering screen

A light filtering screen was chosen to be paired with LEDs due to its light-absorbing features. Light-filtering screens come in a variety of colors to suit a variety of experiments, and work by absorbing all colors except their designated color. For example, a yellow screen would absorb all colors except yellow, providing high contrast between yellow and other colors – creating an ideal environment for a product emitting a yellow fluorescence. This reduces the noise from excitation light to improve observation of the signal of the fluorescence emission. Here, we incorporated 1/8″ thick, 4″ × 2″ acrylic filters, which currently cost ~$0.64 on Amazon.com, into the design (Tables S2 and S3). Acrylic is sold in both transparent and translucent options; users should take note to purchase transparent acrylic only. The utility, affordability, and small size has made it an important component of the Fold-Illuminator. Additionally, we have listed several common fluorescent proteins and dyes, and their recommended LED and acrylic filter wavelengths (Table S3).

### 3.3 ImageJ software

While the intention of the Fold-Illuminator is to enable qualitative assessment of fluorescence, the ImageJ software can allow the Fold-Illuminator to be quantitative for relative comparisons. ImageJ is a software tool that allows for a quantitative evaluation of images [19,27,28]. A user can first image their positive control alongside the experimental reaction with any mobile camera. Then, after downloading ImageJ from the National Institute of Health's website: https://imagej.nih.gov/ij/download.html, a standard curve of fluorophore concentration versus pixel brightness can be established for the positive control. We have included a standard curves of fluorescein isothiocyanate using the Fold-Illuminator and ImageJ in Fig. S1 for reference. Once a standard curve is established, the pixel brightness of the test reaction can be quantified to determine the concentration of the reporter. The utilization of image processing software provides an affordable and portable method for users to quantify fluorescence using the Fold-Illuminator.

## 4 Additional components

### 4.1 USB heater

**Fig. 3:**
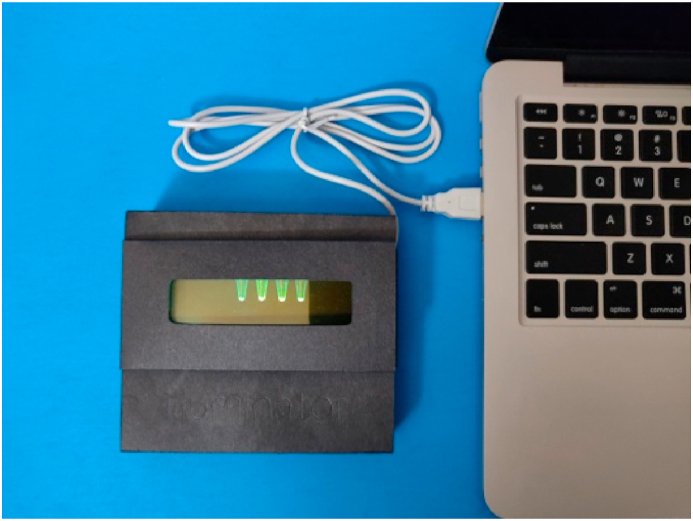
The optional USB heater provides incubation capabilities, offering a possible temperature range of 35–45 **°**C. The USB heater is compatible with any USB-accepting power sources including laptops, power banks, and outlets.

Biochemical processes, such as cell-free protein expression, which produce fluorescent protein products often benefit from incubation at temperatures of ~37 **°**C. Current solutions to providing heat remain divided. In research labs, incubators are commonly used – providing a stable temperature of which the user can set, like a hot version of a fridge. However, these are often expensive pieces of machinery that lack portability. For classroom and field use, a common incubation method is to simply place the reaction in one's pocket. This offers a crude, yet sufficiently warm environment to help promote the reaction of interest. With the goal of providing a more consistent environment for the conduction of biochemical reactions, the Fold-Illuminator has been designed to accommodate an electronic heating element. We opted against chemical heat packs in the spirit of sustainability, as well as to reduce repeat cost of operation. The heating element we have chosen is powered through USB connection (Fig. 3). This design decision no longer makes the Fold-Illuminator a free-standing device; however, this was preferred over the alternate options. In order to make a heating option free-standing, a larger, dedicated battery pack would be required, increasing cost and size. Additionally, we anticipate that the use of the heating element and the LED light would not be simultaneous and controlling both independently in a free-standing device would add complexity to the electronics required. In order to maintain simplicity for the end user, we opted against the use of more complex electronics. We have therefore made the assumption that most users will have access to a laptop or power bank with USB compatibility to support the heating element. USB adapters can also be used to connect the heater to a mobile phone or other portable devices, however, users should be advised to evaluate their device compatibility for such applications. For this reason, we recommend only using laptops, power banks, or outlets to power the USB heater. The selected heating element is able to maintain the Fold-Illuminator at a temperature of ~41 **°**C after warming up for 10–15 min, depending on ambient conditions (Fig. S1, Table S4). Users may make modifications to the device to improve the insulating capacity of the Fold-Illuminator or cut additional vents to obtain a lower temperature. Given the modular nature of this design, we are optimistic that improved electronics solutions for heating will emerge in the coming years that could replace our current solution.

### 4.2 Recyclability and durability

The environmental impact of the Fold-Illuminator was also involved in our design process. The impacts of production, transportation, and disposal were one of our primary design constraints for materials used. This was accomplished by designing the entire exterior with paperboard, achieving a total recyclability of 85% by weight. The only non-recyclable components are the electrical factors including the LED-strips, battery, USB heater, and wiring. Additionally, utilization of paperboard as the exterior shell creates a safe, sturdy structure that prevents loss of function upon common user errors, such as dropping.

The exterior of this device is designed with the intention of being disposable after a small number of uses. Electronics such as the battery and LEDs can be repurposed for many applications globally. As a result, the exterior is less durable than alternate options made of plastic. However, if there is a catastrophic failure to the exterior, a new one can be generated quickly and at a minimal cost.

## 5 Applications of the illuminator

The utility of the Fold-Illuminator is well aligned with a broad range of applications in biological research, and education. However, our impetus was to support the field and classroom applications of cell-free biotechnology, or furthermore to support *in vitro* transcription and translation in the hands of non-experts. Here, we note a few cases that demonstrate a need for the Fold-Illuminator.

Currently, devices such as the transilluminator are abundant in classrooms to enable students to experience hands-on learning of biological concepts, using fluorescence to observe the molecular world. For example, a college biochemistry class at Bandung Institute of Technology in Indonesia used UV transilluminators in a lab exercise involving enzyme preparations to view indication of fibrinolytic activity (Fibrinolysis is a bodily process that prevents blood clots from forming) [29]. The transilluminators detected the glow from fluorescent dye and indicated a complete reaction, allowing for hands-on learning of previously lecture-only material. The use of cell-free systems to teach the genetic code in a learn-by-doing manner is also emerging [5,9,28]. In this process, students are able to learn the fundamental concepts of transcription and translation in an engaging, hands-on manner. Additionally, students who performed this lab showed significant score increases in learning gains compared to the control group [5].

The Fold-Illuminator is a useful tool for indicating when a vaccine has been successfully produced with iVAX, an *in vitro* bioconjugate vaccine expression system [4]. iVAX produces multiple individual doses of vaccines that protect against bacteria such as various strains of *E. coli,* offering a portable do-it-yourself system for the on-demand synthesis of a variety of vaccines. These vaccines are often developed to produce a fluorescent output, allowing for faster visualization through the illumination capabilities of the Fold-Illuminator. iVAX also does not require very cold temperatures during transportation - fitting in with the portability of the Fold-Illuminator as reagents can easily be disseminated for field use. As a highly affordable and portable technology, the Fold-Illuminator thus increases the number of people in LMICs that have access to on-demand vaccine and therapeutics production.

Another field of biology that possesses a need for a portable fluorescence device is in pathogen detection. Current detection methods generally rely on the use of PCR or isothermal amplification to amplify nucleic acids, in turn generating quantifiable concentrations of sample. A requirement of these biological tools is a heat source to promote nucleic acid denaturation, and an acrylic filter with a colored LED can prove beneficial when amplification is paired with a fluorescent output. However, while isothermal amplification methods require a constant temperature (Recombinase polymerase amplification, for example, operates best between 37 and 42 **°**C), PCR requires isothermal cycling – a feature not offered by the Fold-Illuminator [30]. For this reason, the Fold-Illuminator is well-suited for pathogen detection methods that utilize isothermal amplification paired with a fluorescent output. This application is especially pertinent to global health efforts due to recently developed isothermal amplification detection methods of SARS-CoV-2 [31,32].

## 6 Conclusion

Here, we reported the development of an incubator-illuminator device, the Fold-Illuminator. The Fold-Illuminator, when assembled, is a 4.5 × 4 × 1-inch paper-based structure composed of an LED-strip for detection of fluorescence, a light filtering screen for suppression of background fluorescence, and an optional USB heater capable of producing a temperature of ~41 °C. The entire design can then be disassembled back into its original components, offering a high level of portability. At a cost of $5.58, with an optional heating element for an additional $3.98, the Fold-Illuminator offers an affordable device that nearly any user can gain access to.

The Fold-Illuminator is aimed at providing a tool for the incubation of biochemical reactions and detection of fluorescent reporters, supporting applications ranging from education to point-of-care diagnostics. The design provides the user with an easy to assemble, inexpensive, customizable, portable, durable, and recyclable device best suited for education and field-based applications of molecular and synthetic biology. The Fold-Illuminator supports hands-on STEM-based learning in classrooms around the world, and also supports diagnostics applications globally. With increased access to fluorescence visualization and relative quantification, new users and emerging field applications will be able to incorporate fluorescent reporters into their own education and research needs.

### 6.1 Safety considerations and standards

The Illuminator contains an optional heating element composed of electrical wiring, and a 9 V battery to power the LEDs. The USB heater is capable of producing temperatures of approximately 41 **°**C, resulting in the unlikely possibility for small burns or electrical shocks. Additional operational safety information can be found in Supplementary Material 5.

## Ethics approval

This article does not contain any studies with human participants or experimental animals performed by any of the authors.

## Funding

This work was supported by the Baker-Koob Endowment, Bill and Linda Frost Fund, Center for Applications in Biotechnology's Chevron Biotechnology Applied Research Endowment Grant, and the 10.13039/100000001National Science Foundation (NSF-1708919).

## CRediT authorship contribution statement

**Logan R. Burrington:** Writing – original draft, Visualization, Data curation. **Emran Baryal:** Software, Investigation, Resources, Data curation, Writing – review & editing, Funding acquisition. **Katherine Hui:** Software, Investigation, Resources, Data curation, Writing – review & editing, Funding acquisition. **Emmett Lambert:** Software, Investigation, Resources, Data curation, Writing – review & editing, Funding acquisition. **Sarah T. Harding:** Methodology, Supervision, Project administration, Funding acquisition. **Javin P. Oza:** Conceptualization, Supervision, Project administration, Funding acquisition.

## Declaration of competing interest

The authors declare that they have no conflicts of interest.

## Footnotes

Appendix A Supplementary data to this article can be found online at https://doi.org/10.1016/j.synbio.2021.04.003.
