# Supplementary material for: The Fold-Illuminator: A low-cost, portable, and disposable incubator-illuminator device
Source: Synth Syst Biotechnol. 2021 Apr 23;6(2):95–101. doi: 10.1016/j.synbio.2021.04.003 (PMC8099501; doi:10.1016/j.synbio.2021.04.003)
Supplement: Multimedia component 1 [file mmc1.pdf]

The Fold-Illuminator: A Low-Cost, Portable, and Disposable Incubator-Illuminator Device

Logan R. Burrington[1#], Emran Baryal[2#], Katherine Hui[2#], Emmett Lambert[2#], Sarah T. Harding[2], Javin P. Oza[1*]

1 Chemistry and Biochemistry Department, College of Science and Math, California Polytechnic State University, San Luis Obispo, CA 93407

2 Mechanical Engineering Department, College of Engineering, California Polytechnic State University, San Luis Obispo, CA 93407

[#] Authors contributed equally

* Correspondence: joza@calpoly.edu; 805-765-2265

Supporting Information:

Supplementary Figures S1-S2 2-3

Figure S1: ImageJ standard curve using FITC 2

Figure S2: Time course analysis of temperature using the heating element 3

Supplementary Tables S1-4 4-10

Table S1: Fold-Illuminator comparison to other incubators/illuminators 4-5

Table S2: Fold-Illuminator components purchase cost from manufacturers 6-7

Table S3: Fold-Illuminator cost per unit 8

Table S4: Common fluorescence pairings 9

Table S5: Illuminator heating component temperature production versus time 10

Fold-Illuminator Assembly Instructions 11-30

Fold-Illuminator Design 31-37

The Cricut 38-49

Operation and Safety 50

A

B

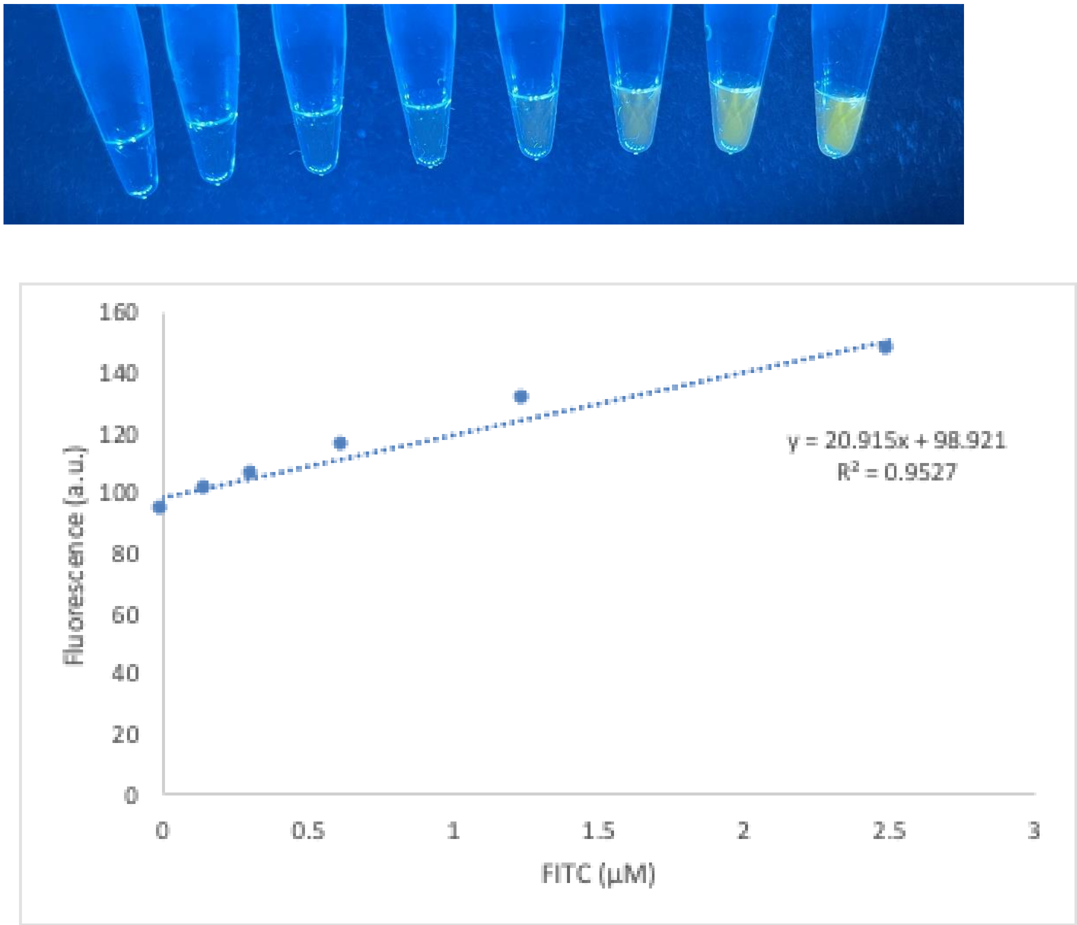

Figure S1: Standard curve of fluorescein isothiocyanate (FITC) using the Fold-Illuminator with Blue LED light and a yellow acrylic filter. Image was captured with an iPhone (A) and processed using (ImageJ) (B).

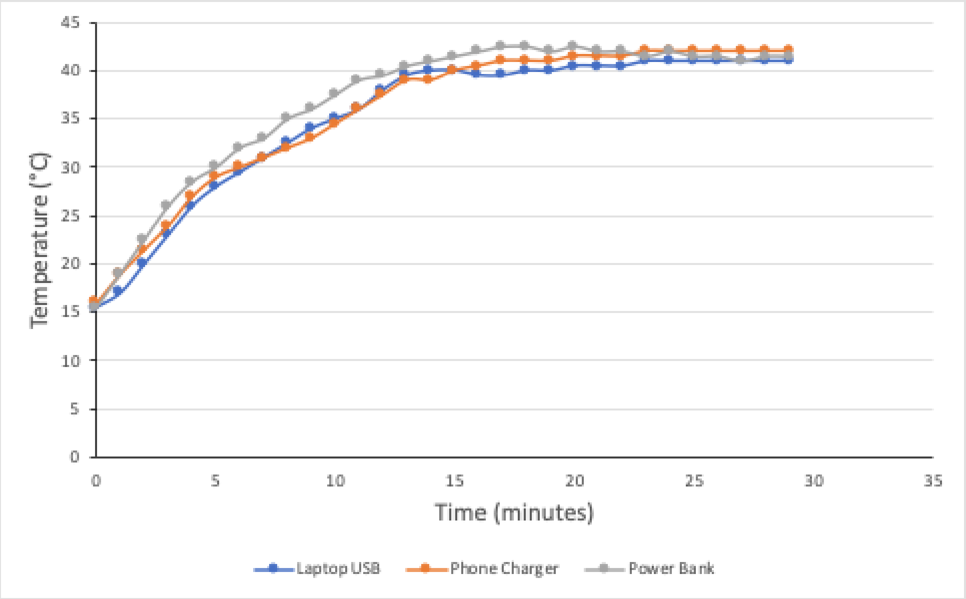

Figure S2: Time course analysis of temperature inside the Illuminator with the additional heating element after plugging into a 2015 Macbook Pro USB (blue), an iPhone charger (orange), and a 5 Volt, 2 Amp iphone charging power bank (gray). Temperatures estimated by placing a thermometer into the center tube holder, avoiding direct contact with the heat pad.

Table S1: Fold-Illuminator Comparison to Other Incubators/Illuminators

Device	Price	Limit of Detection (fluorescein)	Accommodates	Components	Detection Type	Portable	Source*
Fold-Illuminator	$5.58-$9.56	0 - 2.5 µM	PCR tubes, 1.5 mL microfuge tubes	LEDs, light-filtering screen, incubator	Qualitative	Y	
NeoNurture	<$200	-	Infants	Incubator	-	Y	11
ROSALIND	$8.69	0-6.25 µM	PCR tubes	LEDs 3D printed	Qualitative	Y	8
Low-cost fluorescence reader with Cas13a	<$15	6.8 nM	Paper-spotted	LEDs, filter foil, light dependent resistor	Qualitative	Y	24
BioBits Bright portable imagers and incubators	<$100 per 30-person classroom	-	PCR tubes, 96-well plate	Blue or black LEDs, incubator	Qualitative	Y	9
miniPCR P51 Molecular Fluorescent Viewer	$28	-	PCR tubes	LED strip, yellow/orange filter, 9V battery	Qualitative	N	33
Accuris E3000 UV Transilluminator	<$1000	-	Gels	UV Light bulb	Qualitative	N	22
Crystal Technologies Handheld UV Lamp	$530	-	Any	UV Light bulb	Qualitative	N	34
BioTek Cytation 5 Cell Imaging Multi-	>$61000	-	96/384 Well plates	Excitation and Emission filters, any wavelength	Quantitative	N	18

Mode Reader				light bulb, Incubator			

* reference list matches main text

Table S2: Fold-Illuminator Components Purchase Cost from Manufacturers

Item	Quantity	Vendor	Manufacturer	Model number	Price
490nm Blue LED strip	64	Waveform lighting	-	-	$130.69*
9V battery	8	Amazon	AmazonBasics	6LR16-8PK	$10.99**
9V battery holder	5	Amazon	LAMPVPATH	ASIN: B07BXBS93X	$8.99**
4-ply (22”x28”) railroad board	50	Amazon	ROSELLE PAPER INC	1485728	$11.92**
Filter sheet	18	Amazon	AcrylandUSA	ASIN: B006FLYKF4	$10.99**
Velcro dots	250	Amazon	Strenco	VD34W 250	$11.48**
LED to wire connector	10	Amazon	QIJIE	NLCSEN10XB2	$8.98**
Heating element	1	Ebay	-	-	$3.89**
Cricut	1	Cricut	Cricut	-	$369.99
Cricut StandardGrip Machine Mat, 12”x24”	2	Cricut	Cricut	-	$17.19
Cricut Deep Cut Blade and Housing	1	Michaels	Cricut	D032562S	$34.99

Item	Link to Purchase
490 nm Blue LED strip	https://www.waveformlighting.com/datasheets/CS_7021.pdf
9V batter holder	https://www.amazon.com/LAMPVPATH-Pack-Battery-Holder-Switch/dp/B07BXBS93X
4-ply (22”x28”) railroad board	https://www.amazon.com/School-Smart-1485728-Railroad-Thickness/dp/B00PEFCCJ8
Filter sheet	https://www.amazon.com/Cast-Acrylic-Sheet-Yellow-Nominal/dp/B006FLYKF4
Velcro dots	https://www.amazon.com/Hook-Loop-Dots-White-Sets/dp/B01M5ASDBH
LED to wire connector	https://www.amazon.com/gp/product/B01MT50ZCZ
Heating element	https://www.ebay.com/itm/5V-Portable-USB-Heating-Heater-Winter-Warm-Plate-For-DIY-Shoes-Gloves-Pad-Mat/173788708266
Cricut	https://cricut.com/en_us/machines/cricut-maker/cricut-makerr-machine-champagne.html#product_detail
Cricut StandardGrip	https://cricut.com/en_us/standardgrip-machine-mat-12-x-24-2-ct.html

Machine Mat, 12”x24”	
Cricut Deep Cut Blade and Housing	https://www.michaels.com/cricut-explore-deep-cut-blade-and-housing/D032562S.html

* Tax not included

** Tax included

Table S3: Fold-Illuminator Cost per Unit

Portable Fluorescence Illuminator
Indented Bill of Material (iBOM)

Assembly Level	Part Number	Description	Qty	Cost	Ttl Cost	Source	More Info
		Lvl0 Lvl1 Lvl2 Lvl3 Lvl4					
0	100000	Final Assy				------	
1	111000	Outer Assembly				------	
2	111100	Sheath	1	0.13	0.13	Custom	Paperboard, cut from 11"x 24"
2	111200	Orange Light Filter	1	0.64	0.64	Amazon	Yellow Acrylic, 1/8" thick, 4" x 2"
2	111300	Box	1	0.08	0.08	Custom	Paperboard, 11"x 24"
3	111310	Test Tube Stand				------	
4	111311	Small Test Tube Stand	1	0.03	0.03	Custom	Paperboard, 11"x 24"
4	111312	Large Test Tube Stand	1	0.03	0.03	Custom	Paperboard, 11"x 24"
3	111320	Electrical Components				------	
4	111321	Blue LED light strip	1	1.45	1.45	Waveform Lighting	Blue LED light strip, 10 mm width, 3 inch length
4	111322	USB Heater	1	3.98	3.98	Ebay	5V, carbon fiber, 3.74" × 2.56"
4	111323	Battery	1	1.38	1.38	Amazon	9V
4	111324	Battery Housing	1	0.85	0.85	Amazon	9V snap connection with switch
4	111325	LED to Wire Connector	1	0.90	0.90	Amazon	10 mm LED strip, plastic
2	111400	Attachments				------	
3	111410	Velcro Dots (pair)	3	0.03	0.09	Amazon	0.75" diameter Velcro dots
	**Total Parts**		**13**		**9.56**		

Table S4: Common Fluorescence Pairings

Fluorescent Protein/Dye	Fluorescence Brightness	Excitation wavelength(nm)	Acrylic Filter color	Source
Blue Fluorescent Protein (mTFP1)	54.4	460	Yellow/Orange	DOI: 10.1038/s41592-019-0352-8
Green Fluorescent Protein (sfGFP)	54.2	480	Yellow/Orange	DOI: 10.1038/s41592-019-0352-8
Yellow Fluorescent Protein (YPet)	80.1	520	Orange/Red	DOI: 10.1038/s41592-019-0352-8
Red Fluorescent Protein (mCherry)	15.8	580	Blue/Purple	DOI: 10.1038/s41592-019-0352-8
Fluorescein	~65	475	Yellow/Orange	J.R. Lakowicz, Principles of Fluorescence Spectroscopy, 2nd Ed., Kluwer Academic/Plenum Publishers, New York, London, Moscow, Dordrecht, 1999.
β-galactosidase (with oNPG)	N/A	White Light	None	DOI: 10.1002/pro.2165
XylE (with catechol)	N/A	White Light	None	DOI: 10.1128/jb.171.12.6617-6624.1989

- All colors and wavelengths are our recommendations for pairing with the respective fluorescent protein or output.

Table S5: Illuminator Heating Component Temperature Production Versus Time

Time (min)	Temperature (°C)		
	Laptop USB	Phone Charger	Power Bank
0	15.5	16	15.5
1	17	19	19
2	20	21.5	22.5
3	23	24	26
4	26	27	28.5
5	28	29	30
6	29.5	30	32
7	31	31	33
8	32.5	32	35
9	34	33	36
10	35	34.5	37.5
11	36	36	39
12	38	37.5	39.5
13	39.5	39	40.5
14	40	39	41
15	40	40	41.5
16	39.5	40.5	42
17	39.5	41	42.5
18	40	41	42.5
19	40	41	42
20	40.5	41.5	42.5
21	40.5	41.5	42
22	40.5	41.5	42
23	41	42	41.5
24	41	42	42
25	41	42	41.5
26	41	42	41.5
27	41	42	41
28	41	42	41.5
29	41	42	41.5
30	41	42	41.5

Fold-illuminator Assembly Instructions

Assembly video available at: https://www.youtube.com/channel/UCoxxTKNHAJlapybWZ8iRnKw

Preliminary Check

Make sure you have all the listed Illuminator components in your kit.

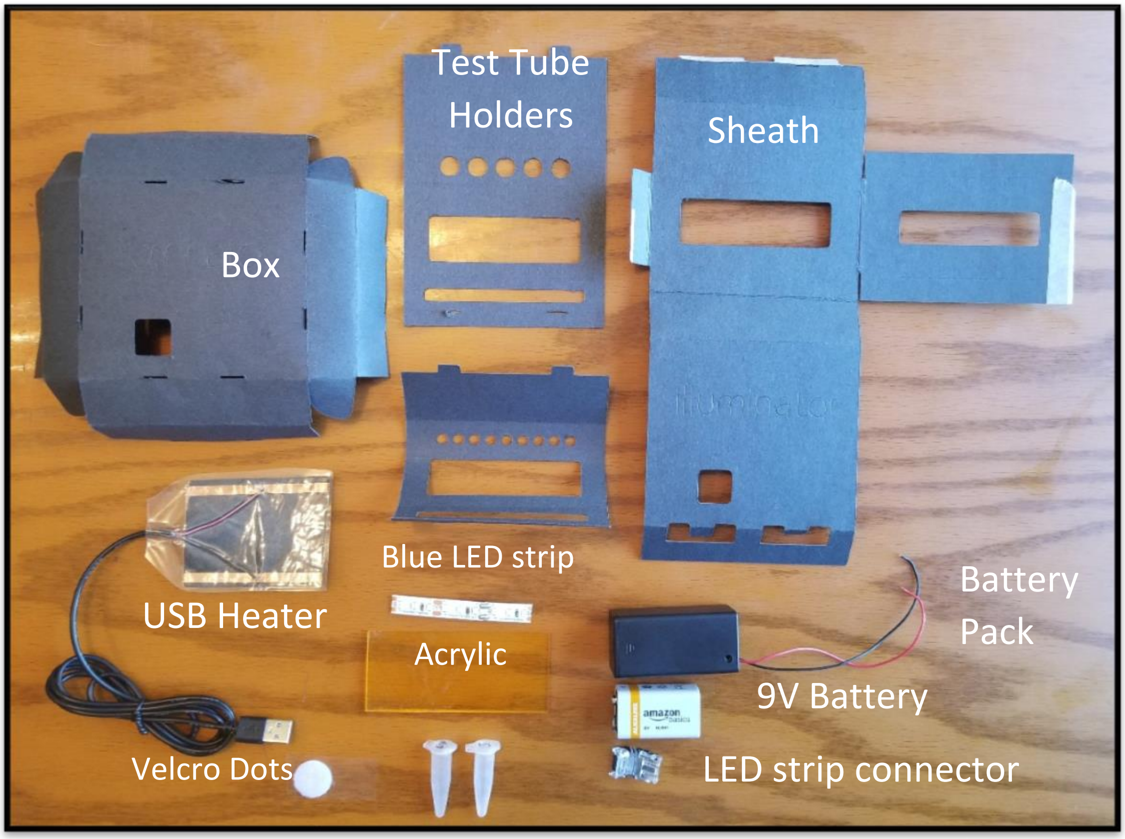

Description	Checklist
(1) Sheath Piece	
(1) Box Piece	
(1) Test Tube Holder, 0.6 mL (9-hole pattern)	
(1) Test Tube Holder, 2.0 mL (5-hold pattern)	
(1) LED Strip to Wire Connector	
(1) 4” × 2” Yellow Acrylic Filter	
(1) 9-Volt Battery	

(1) 9-Volt Battery Holder	
(1) Blue LED Light Strip	
(1) USB Heater Pad	
(2) Velcro Dot Pairs – Hook and loop tape (alternatively)	

1. Box Instructions

1.1 Take the box piece out of the kit. With the "Illuminator" indentation facing downwards and the circled cut-out in the bottom right-hand side as shown in the image, take a ruler or use a table or counter edge to make creases along the pre-scored lines on the paper. Crease along all of the RED lines <u>towards</u> you in the orientation shown.

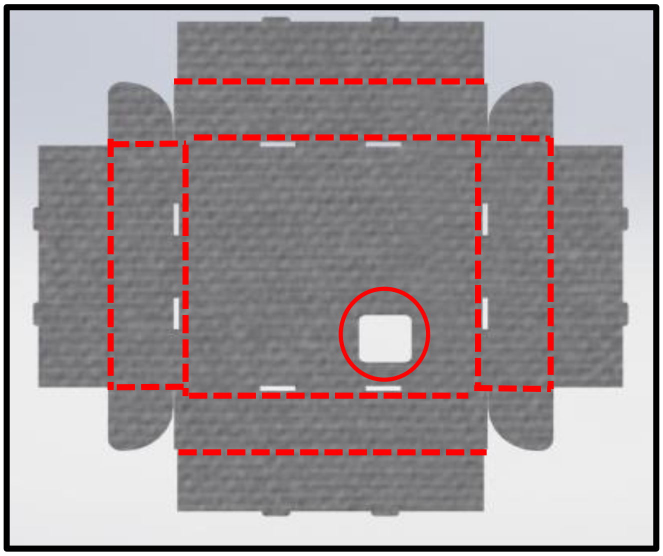

1.2 Next, lay the box piece flat with the side **<u>without</u>** the "Illuminator" indentation facing up. Orient the piece so the square cutout is on the bottom right, as shown in the image below. This orientation will be used when referencing the "top", "bottom", and "side" panels.

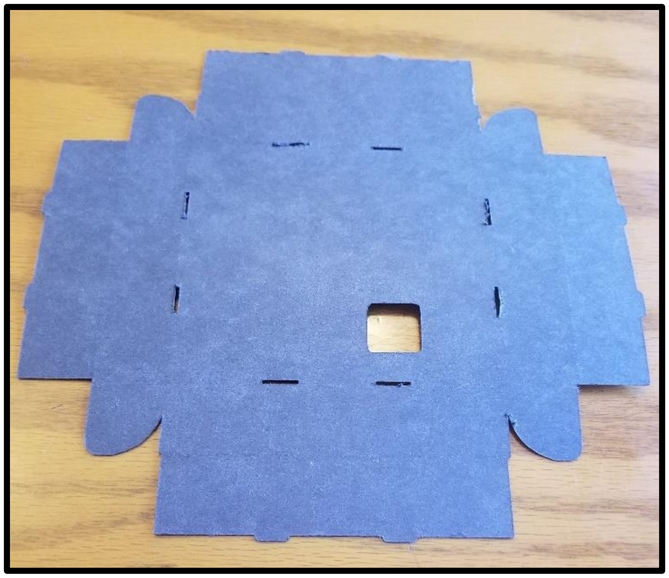

1.3 Fold the left and right panels along the scored lines closest to the center of the box, making the panels vertical.

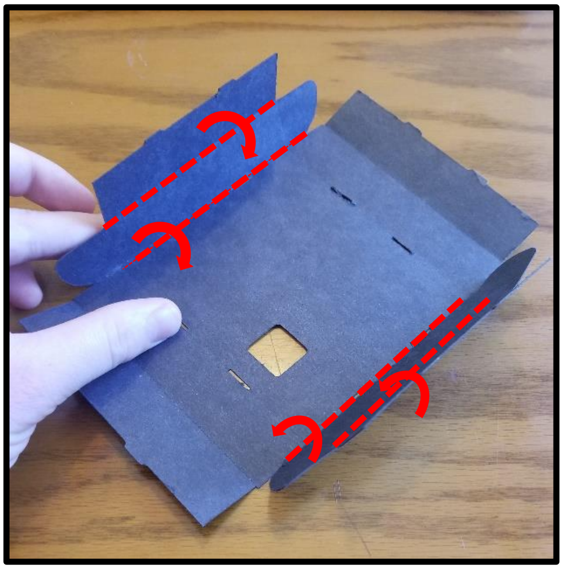

1.4 Fold the rounded tabs on both side panels inward so that the tabs and side panels form a 90° angle with the panels.

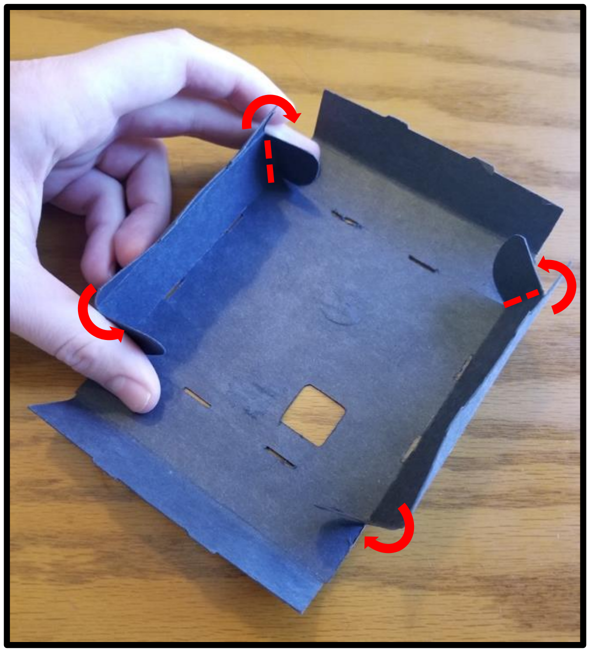

1.5 Fold the top and bottom panels upward along the scored lines closest to the center of the box.

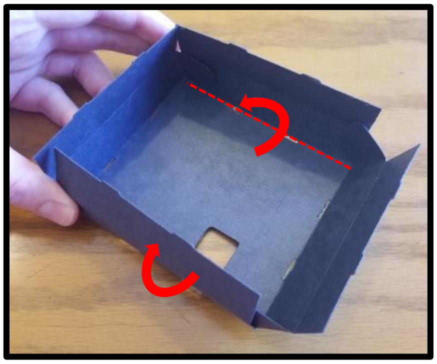

1.6 Fold the top and bottom panels at the crease in the middle over the rounded tabs on the side panels, inserting the small tabs at the panel's ends into the holes in the base of the box.

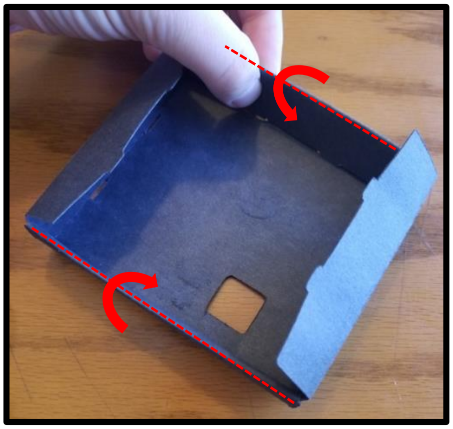

1.7Fold the left and right side panels at the crease in the middle and down, inserting the small tabs at the ends into the holes in the base of the box. The box is now complete.

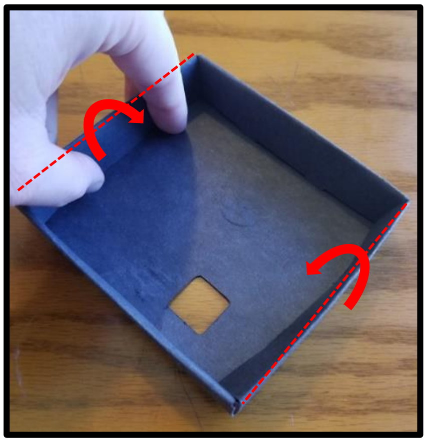

2. Test Tube Holder Instructions

2.1 Take out the small and large test tube holder pieces from the kit and orient them with the tabs pointing upwards, as shown below. Using the same method as before, crease all of the scored lines on the test tube holders inwards from the current view.

*** Be particularly careful folding along the crease with the narrow horizontal cutouts at the bottom of the holder. ***

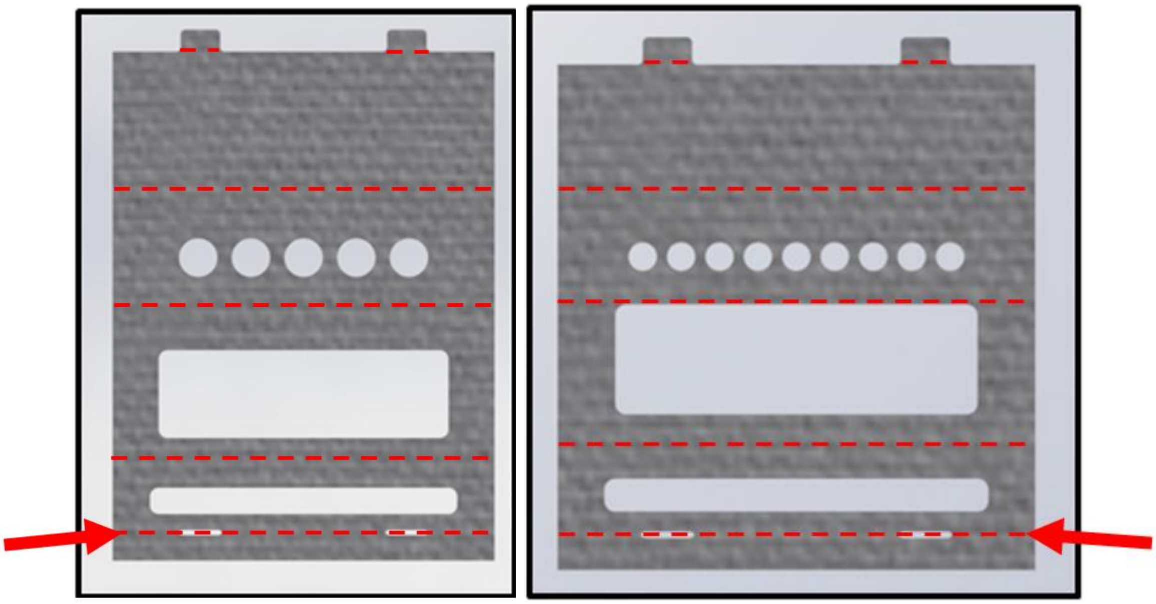

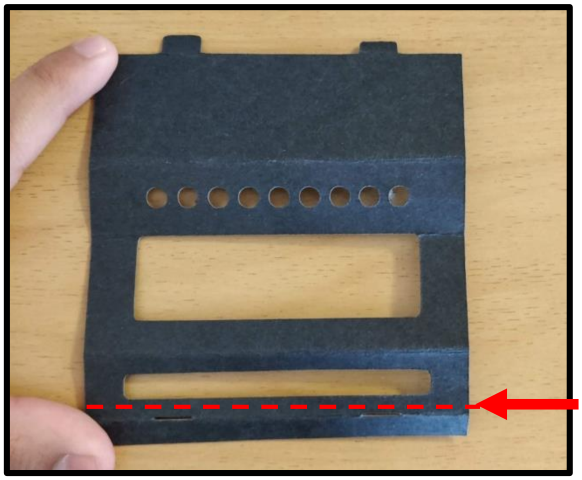

Once done, return the pieces to the orientation below, with the folds pointing away from you.

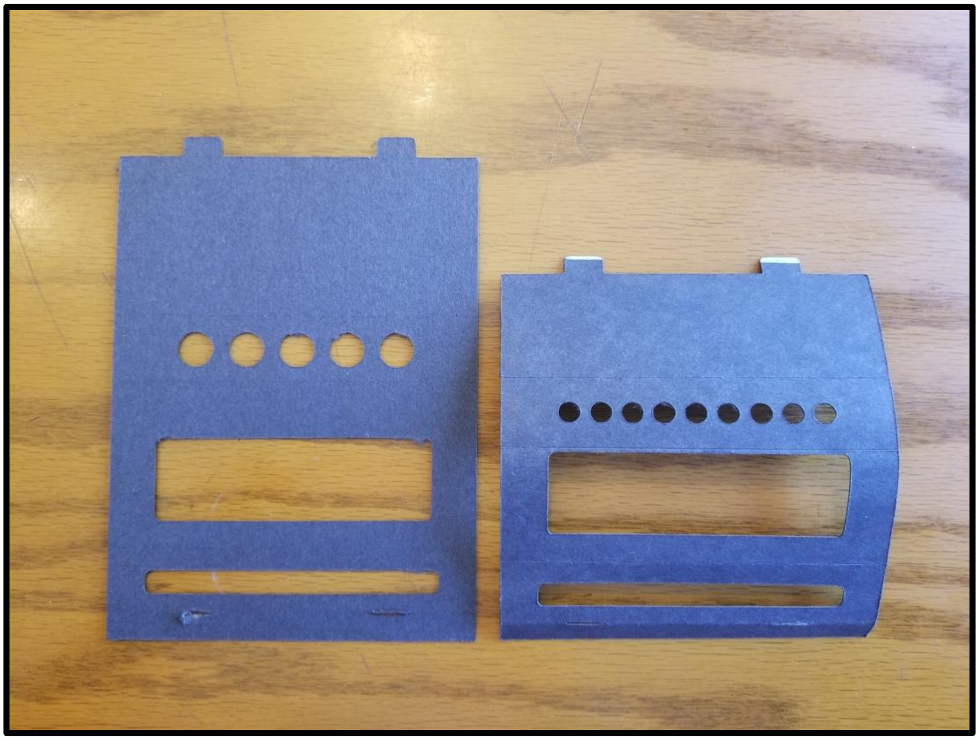

2.2 Fold the box around into a rectangular prism and insert the tabs from the top panel into the narrow horizontal slits at the bottom panel. Make sure to insert the tabs firmly into the slits until they are secure.

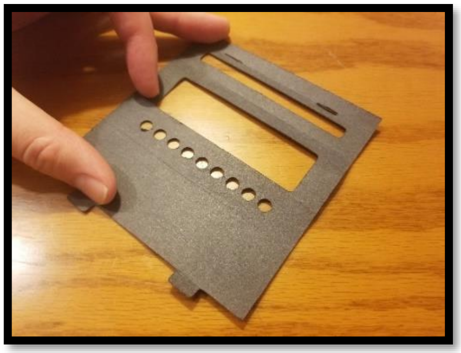

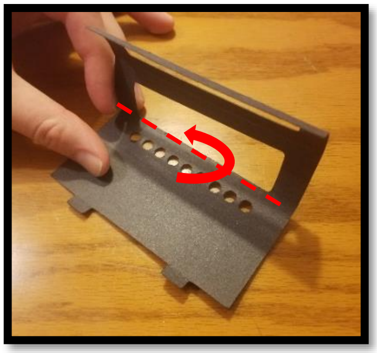

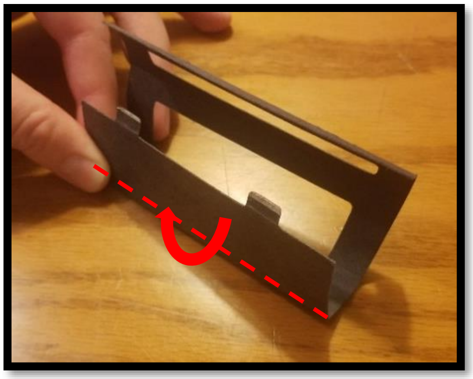
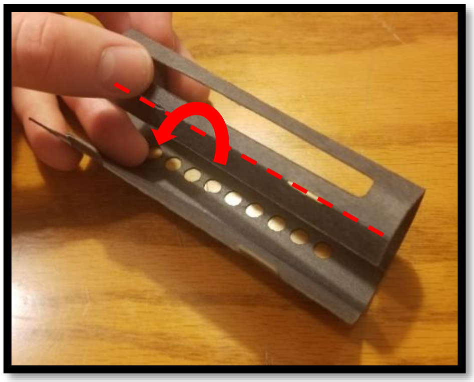
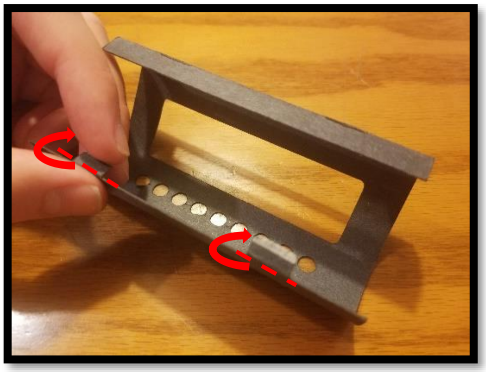
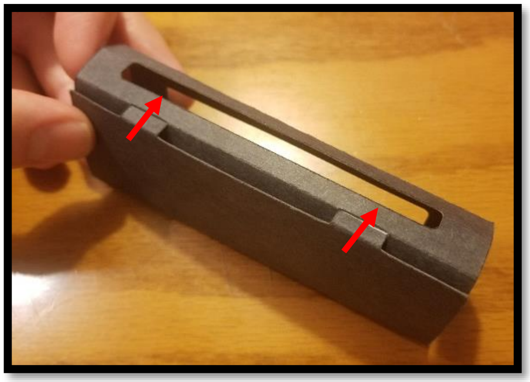
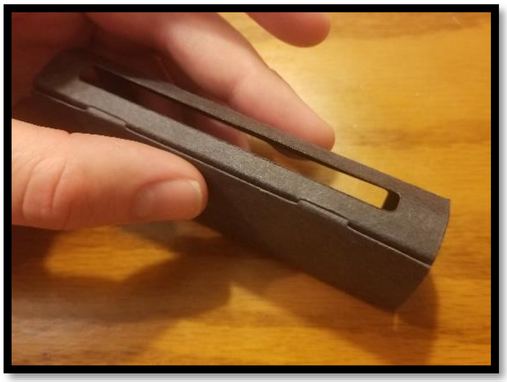
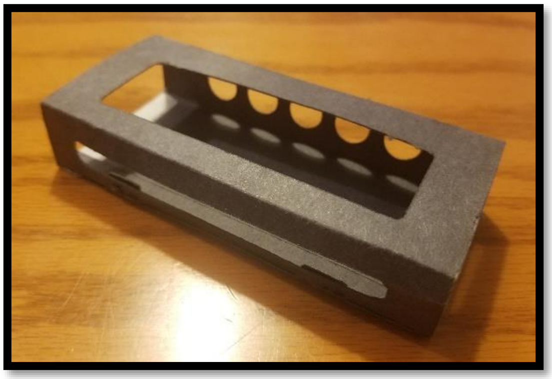

2.3 Assemble the other test tube holder in a similar manner; the test tube holders should be able to stand upright and have a rectangular box prism form when complete.

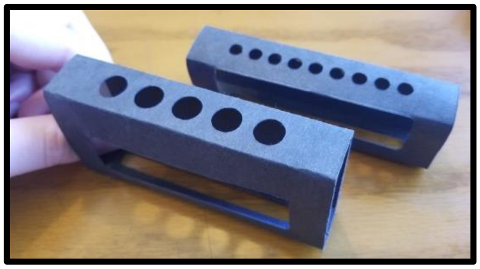

3. Sheath Instructions

3.1 Take out the Sheath piece from the kit. Place the sheath with the “Illuminator” indentation facing down and the light filter piece sticking out to the left, as shown in the image below. Crease along the pre-scored lines as before, folding RED lines towards you. Next, crease the BLUE lines away from you in the orientation shown.

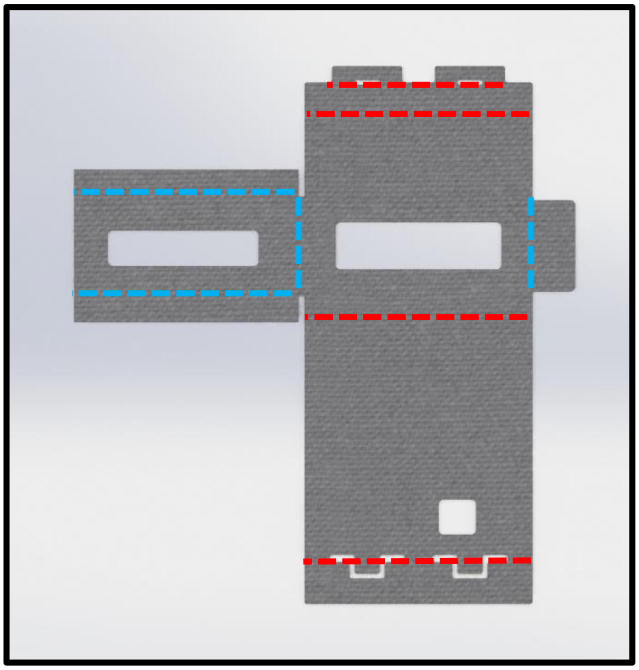

3.2 To assemble the sheath, position it in the unfolded position as shown so that the indented ‘Illuminator’ logo is facing downward and the light filter holder sticks out to the left.

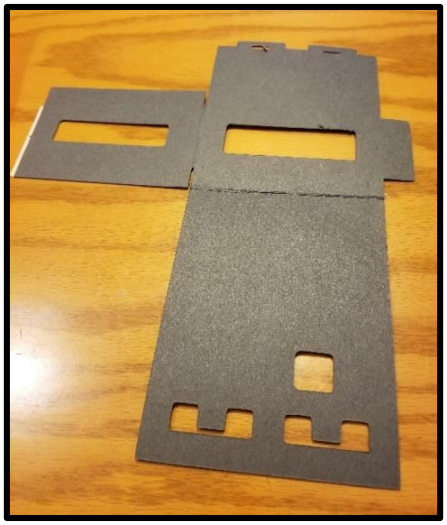

3.3 Take the USB heater and Velcro dots from the kit. Attach a Velcro dot with “loops” (soft) onto the back of the USB heating element and place a Velcro dot with “hooks” (scratchy) on top of the other Velcro dot. Orient the heating element with the USB cable sticking to the right over the panel with the square cut-out, placing it slightly above the cut-out and centered on the panel.

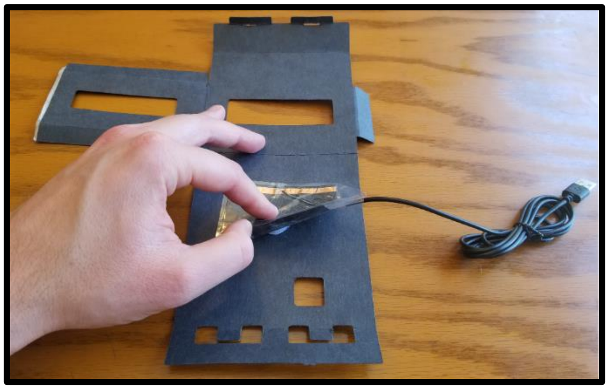

3.4 Fold along the scored line adjacent to the viewing window as shown so that the window is vertical.

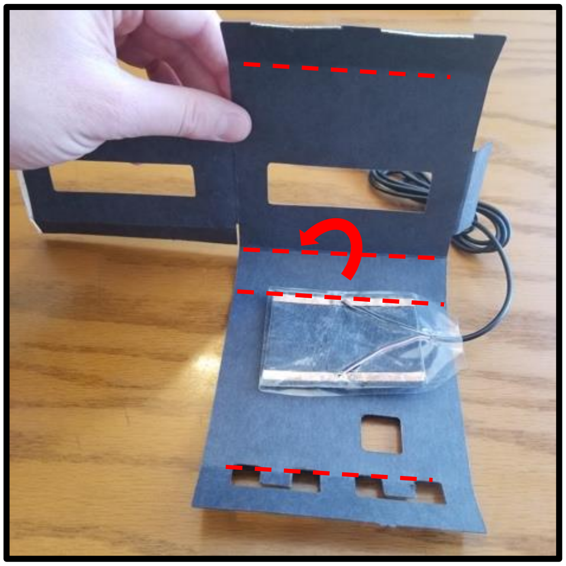

3.5 Fold along the horizontal red fold lines portrayed in step 3.1 so that the sheath closes with the small tabs on the base and top of the sheath close together as shown.

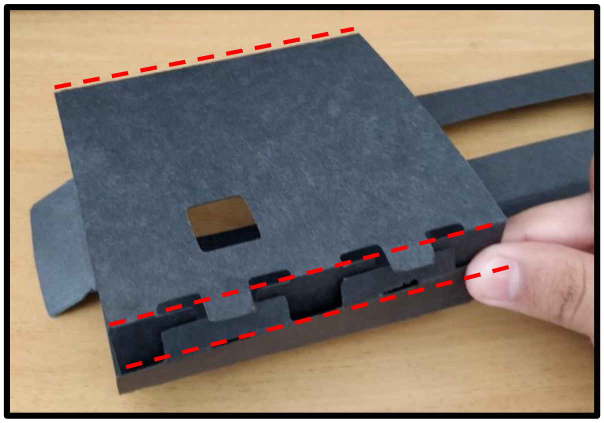

3.6 Insert the wider tabs into the opening as shown below.

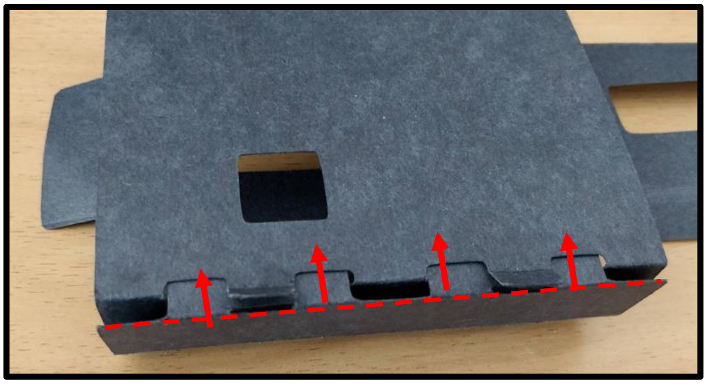

3.7 Now carefully insert the smaller tabs into the opennings within the wider tabs as shown below.

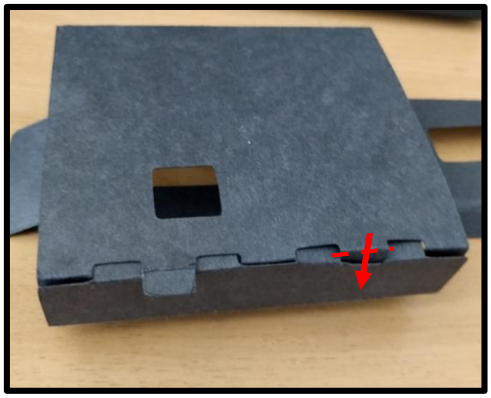

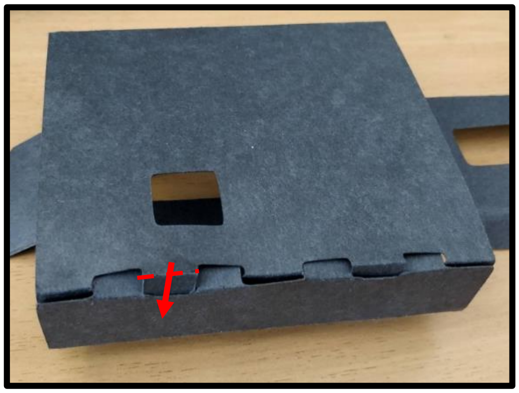

3.8 Push the tabs in further until they're fully secure.

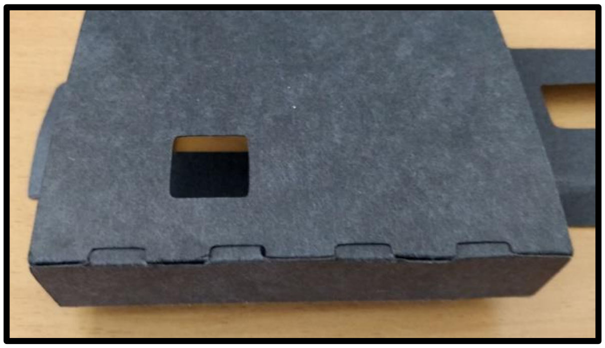

3.9 With the viewing window facing upright and the filter holder out to the left, fold the top and bottom creases on the filter holder. Take the yellow acrylic from the kit and slide it underneath the new top and bottom flaps on the filter holder, as shown below.

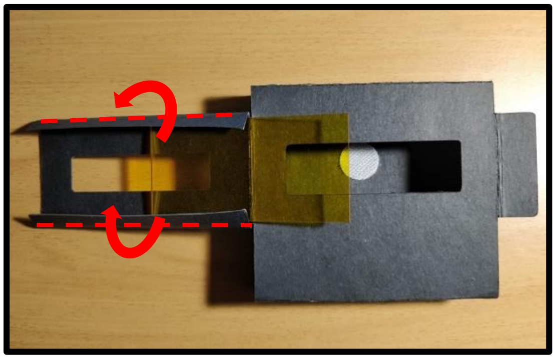

3.10 With the filter fully inserted, flip the filter holder over the viewing window, as shown in the image at lower left. Then insert the side tab into the filter holder in the space front of the acrylic.

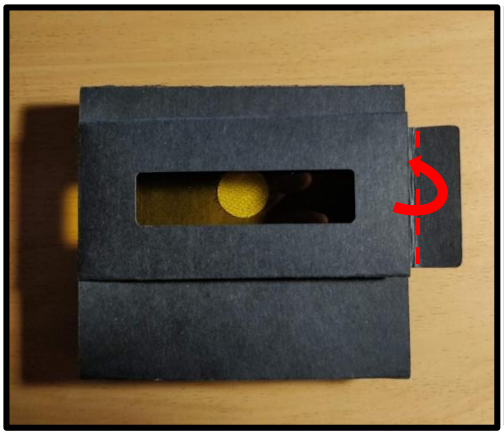

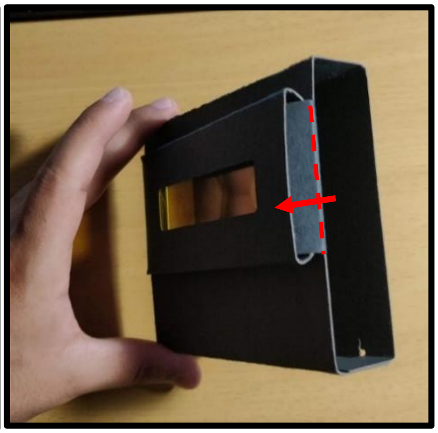

3.11 Pinch along the folds of the filter holder and push the filter further to the left until it sits firmly in place.

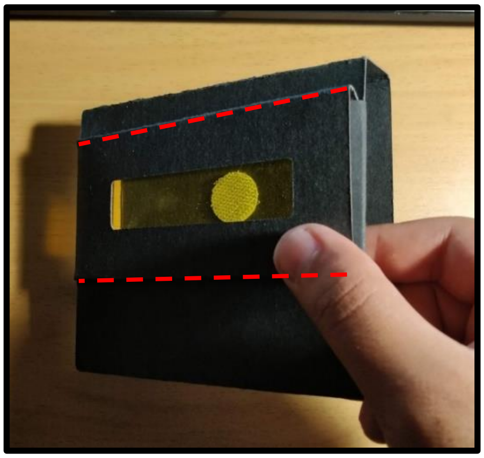

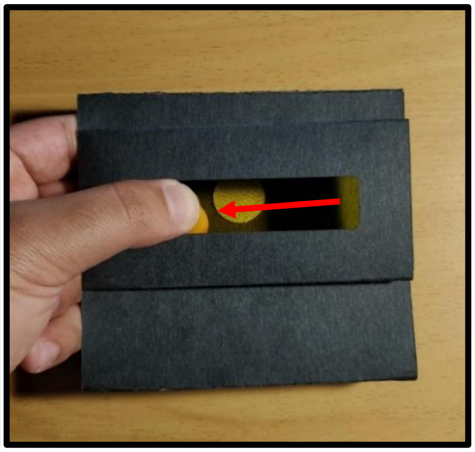

4. Electrical Circuit Assembly

4.1 Take all remaining components out of kit, which should include the 9-Volt battery, battery pack, LED strip, LED to wire connector, and two Velcro dot pairs.

4.2 Ensure the "ON/OFF" slider is on "OFF". Remove cover from battery pack, insert the 9-Volt battery with the proper terminal orientation as shown. It may take some force to snap the battery into place.

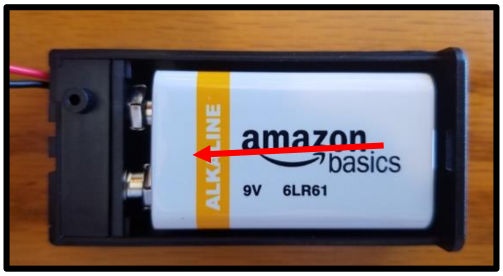

4.3 Remove the red backing from the adhesive tape from underneath the LED strip.

*** Beware of the sharp corners of LED strip. ***

Orient the battery case so the power button is facing away from you and the wires are coming out of the bottom right side. Place the adhesive of the LED strip on the top of the battery pack as shown, with the positive (+) side of the LED strip aligned with the back edge of the battery case (side with the power switch) and the end of the strip aligned with the left side (side without the wires).

Ensure the strip does not block the removable battery lid. Once you confirm that it's not blocked, slide in the battery holder cover.

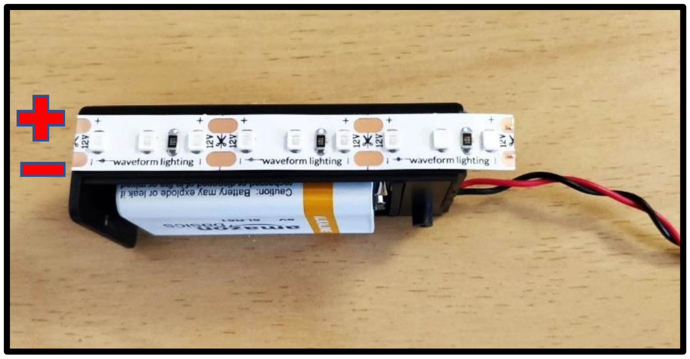

4.4 Use your nails to scratch off adhesive from the hanging portion of LED strip. Remove enough of the adhesive (about 1 cm) so there isn't any under the copper leads of the strip.

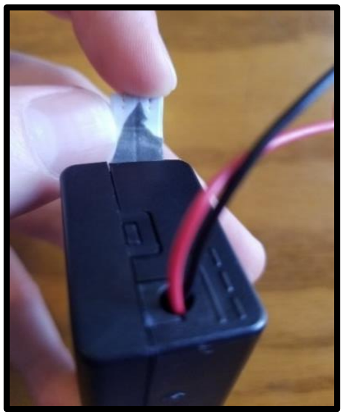

4.5 Flip the cap on the LED to wire connector up until you can completely see the sharp metal leads on the LED strip side. The side with two semicircular indentations in the base is for the wires, and the side with the flat base is for the LED strip, which is shown below.

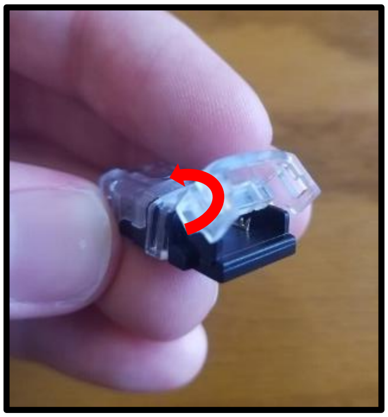

4.6 Insert the hanging end of the LED strip into the connector's LED side, positioning the shiny copper terminals on the LED strip over the metal pins in the connector. This is important, as the pin and LED copper terminal connection will provide battery power to the lights. The pins in the connector should create indentations in the copper LED terminals.

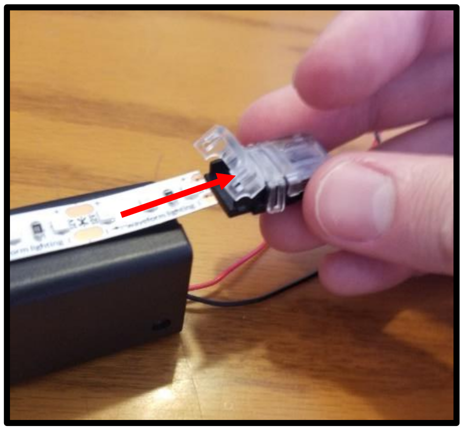

4.7 Ensure that the strip is not too far into the connector and the last LED is clear of the tab on the connector cap before slowly closing the cap. Ideally, the clear cap should snap into place once closed.

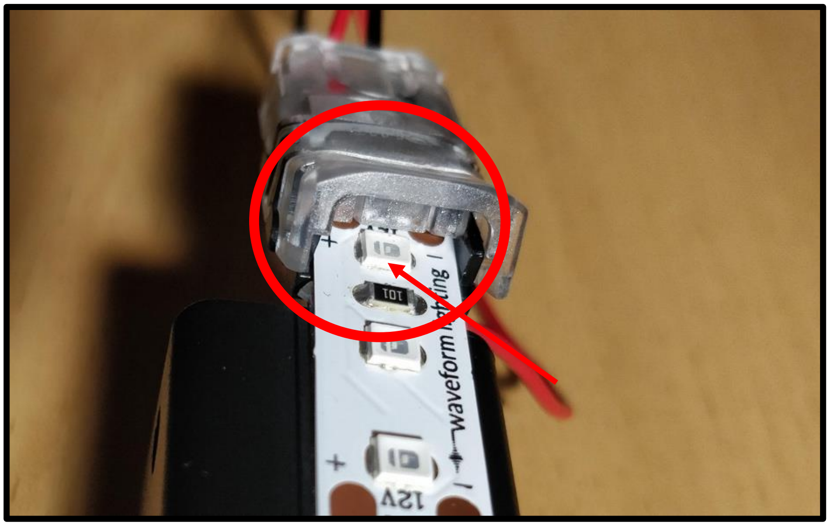

4.8 Turn the opposing white cap up vertically until you see the metallic "V" wire connectors.

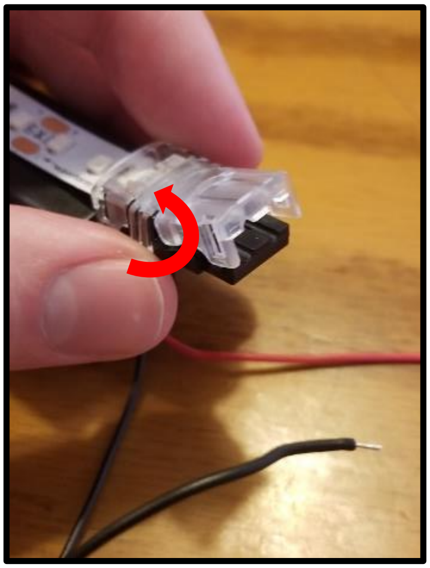

4.9 Insert wires into connector opening so that they rest on the metallic “V” grooves inside the connector. Make sure that the red wire is on the same side of the LED strip that indicates a positive (+) sign (RIGHT). Similarly, the black wire should be on the side of the negative (-) sign on the LED (LEFT).

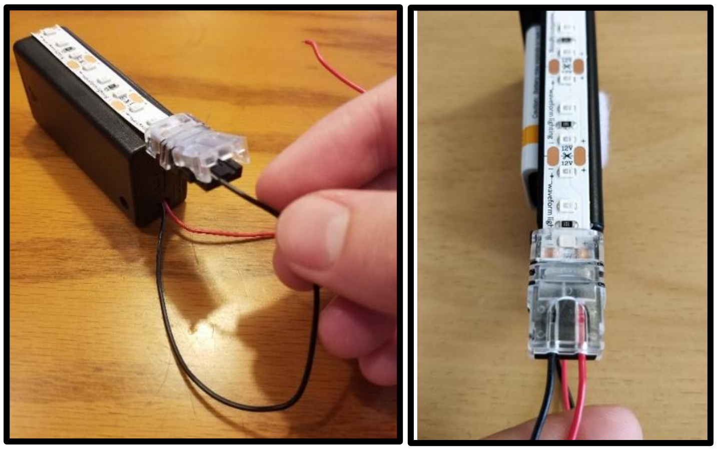

4.10 Once wires are lined up within their respective grooves, close the cap until it snaps into place.

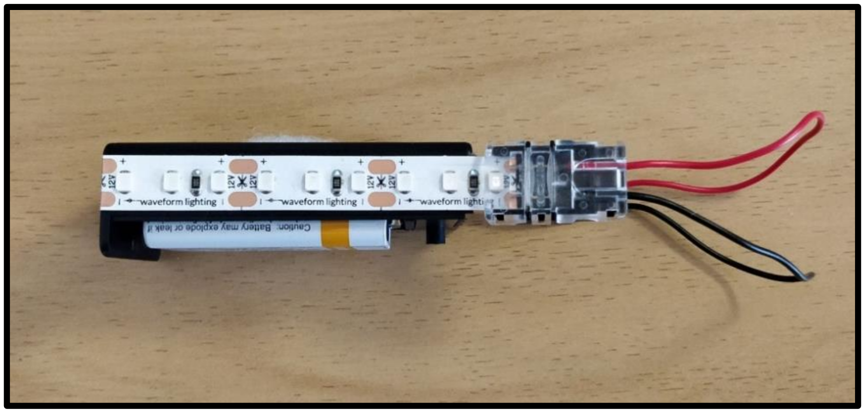

4.11 Once wires are lined up within their respective channel, make sure the metal mart of the wire will come into contact with the metal in the channel, or else the lights will not work. When in place, snap the cap down over the wires.

4.12 Turn on the power button at the back of the battery case to test the lighting.

NOTE: To avoid discomfort due to bright LEDs, point the LEDs away from your eyes when turning them on.

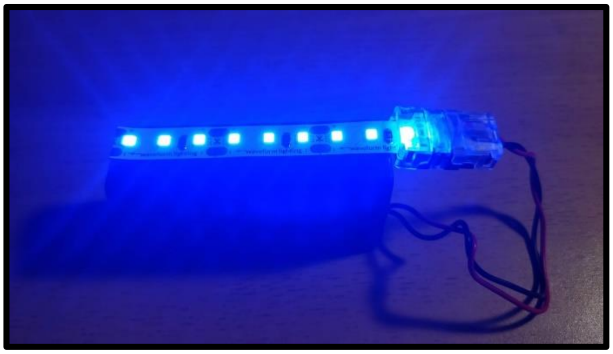

TROUBLESHOOTING: In case the lights do not turn on, do the following

1. Redo the connection between the LED strip and Connector
2. Check the wires and LED strip polarity. Ensure that (+) lines up with the Red wire and (-) lines up with Black.

3. Check the battery connection and polarity. Ensure that the leads properly snap into the leads of the battery housing.

4.13 Attach a Velcro dot with "loops" (soft) behind the switch on the side of the battery pack with the switch. Then place a Velcro dot with "hooks" (scratchy) on top of it, as shown. Hook and loop tape can be used as well.

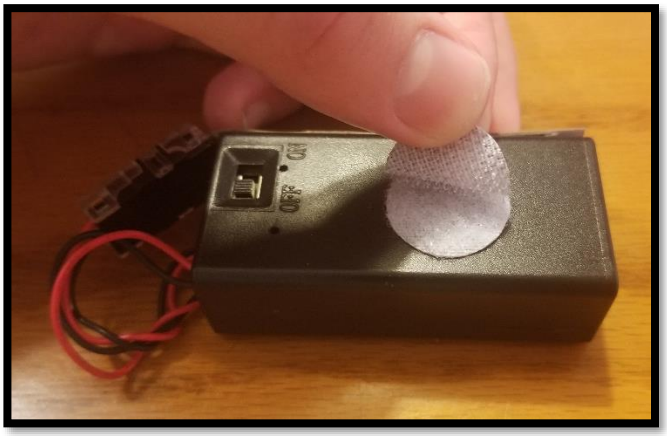

4.14 Line up the power switch on the battery with the cut-out hole at the bottom of the box. Then attach the battery pack and test tube rack to the inside of the box as shown.

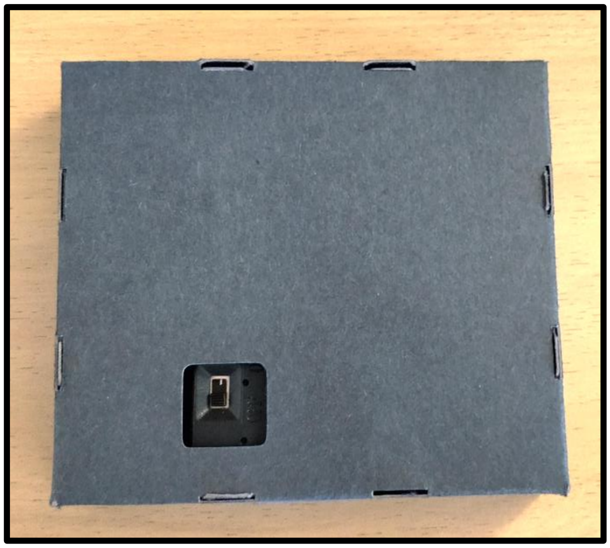

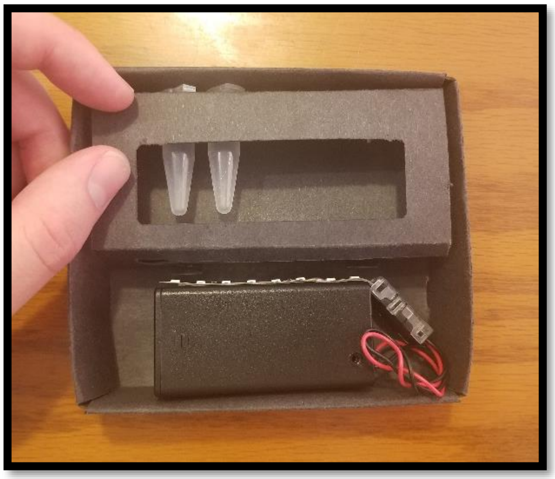

4.15 Finally, slide the box into the sheath. The Illuminator is complete!

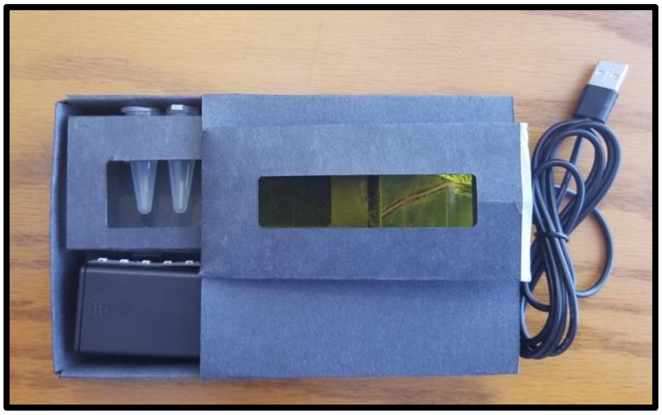

Fold-illuminator Design

Figure 1. New Illuminator Final Design Full Assembly

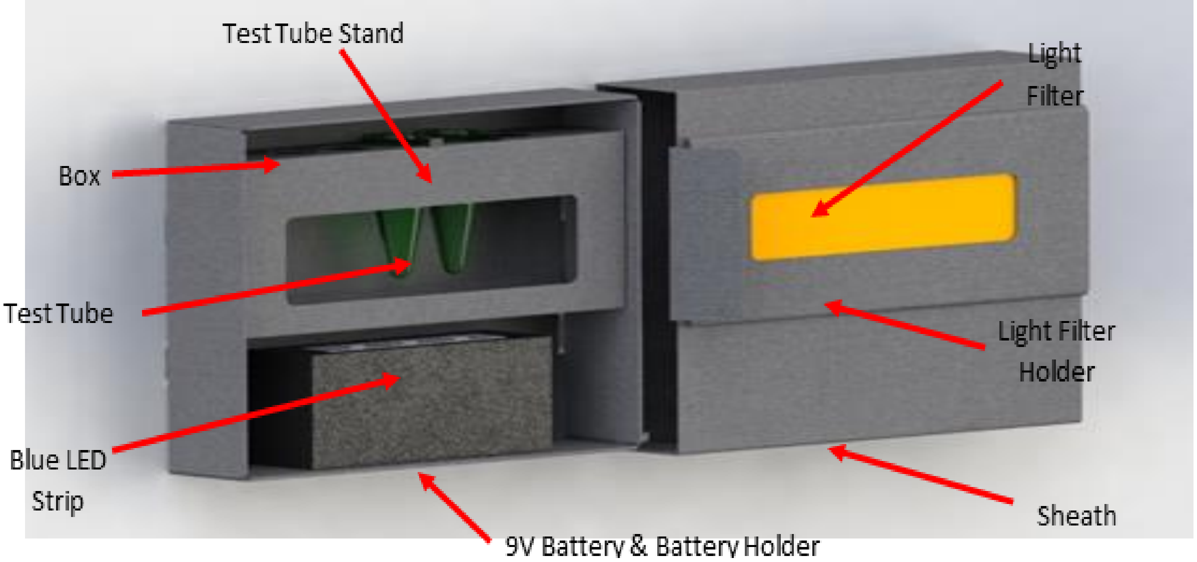

Sheath and Box

The sheath includes a filter holder mechanism and self-locking tabs for the sheath to hold its shape. The dimensions of the sheath are 4.55” × 4.12” × 1.14”.

The sheath’s unfolded and folded isometric views in Figure 2 display the filter holder mechanism and the self-locking tabs. These were both implemented in order to reduce the use of adhesives like tape, glue, or Velcro dots to form the sheath. To assemble the sheath using the self-locking tabs, the sheath is folded at the designated lines, and the tabs on the top are inserted into the slits cut out at the bottom.

Figure 2. Sheath Design

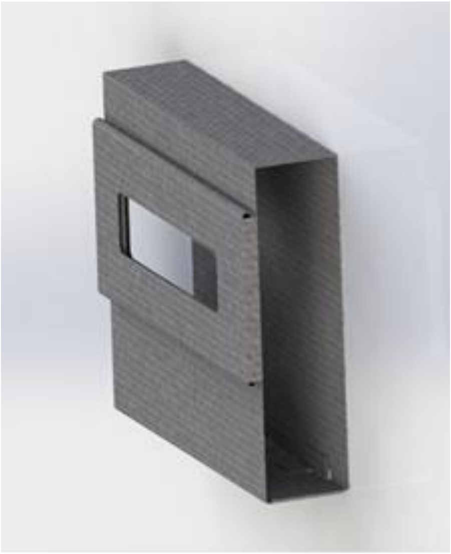

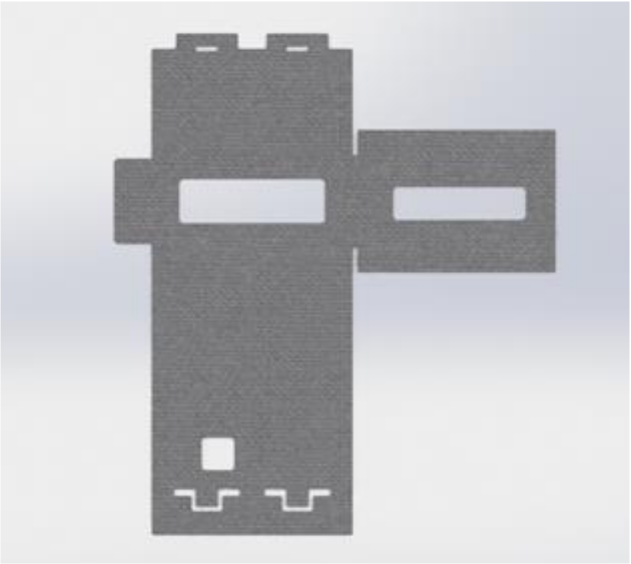

(a) Folded	(b) Unfolded

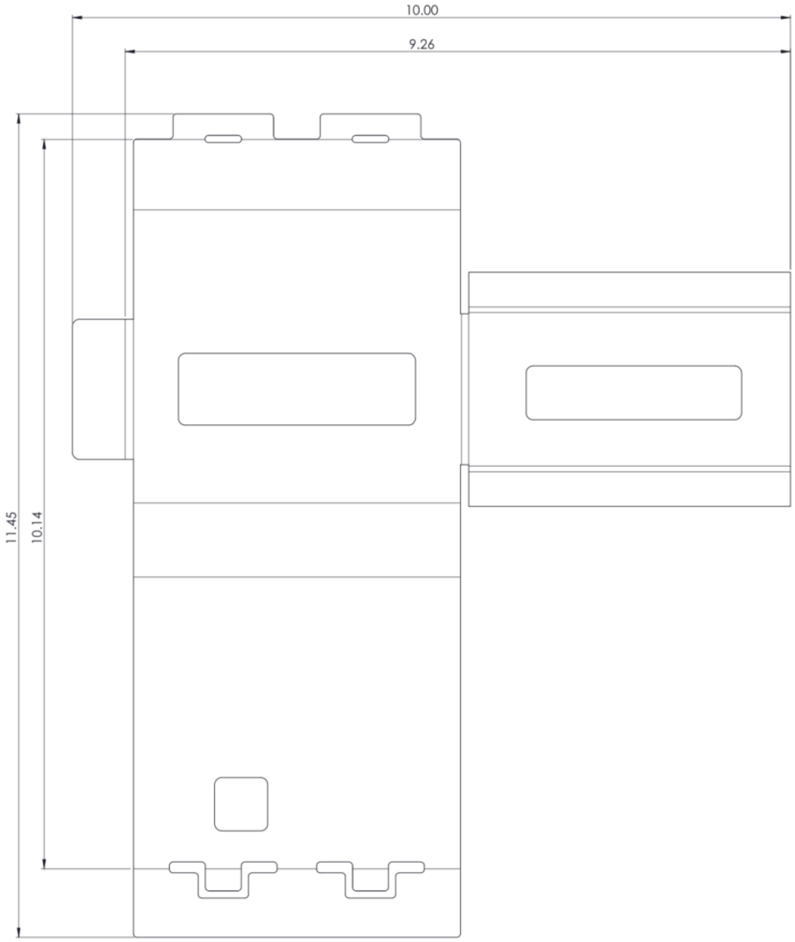

(c) Unfolded with dimensions (inches)

The filter holder mechanism added onto the sheath acts as a pocket for the acrylic panel to be inserted in. A tab on the right inserts into the flaps folded down at the top and bottom to form the pocket, and the acrylic filter sits between the front of the filter holder mechanism and the front of the sheath's viewing window. Thus, the filter can be inserted and removed without using any adhesive attachments and effectively filters out the light to view the fluorescent glow. The filter color is easily interchangable depending on user needs.

The box has a simplified flat pattern and includes tabs to hold the walls of the box up when assembled. The box's dimensions are 4.5" × 4" × 1". The box's flat pattern and folded view is shown in Figure 3.

Figure 3. Box Design

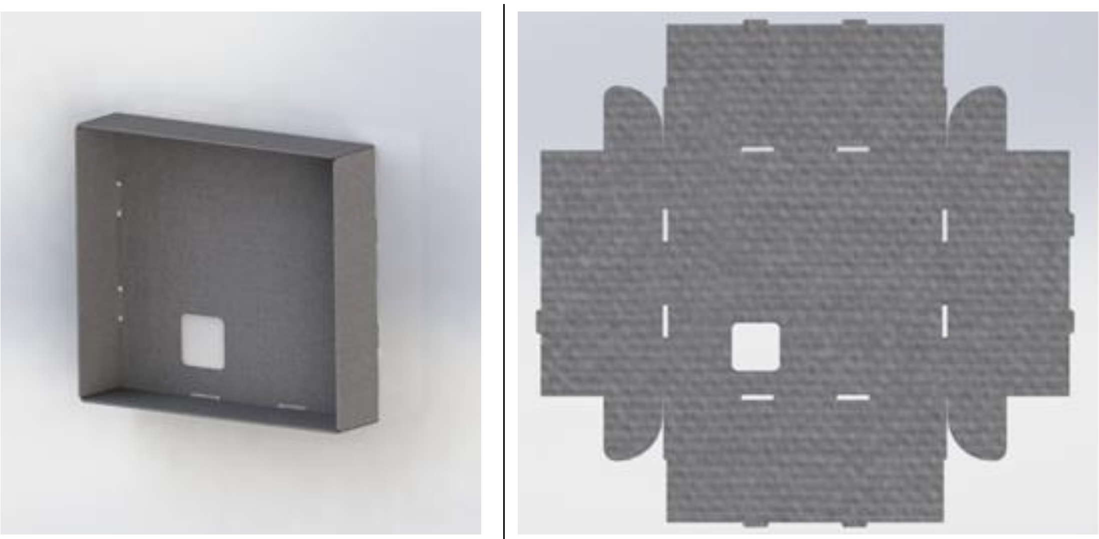

(a) Folded	*(b) Unfolded*

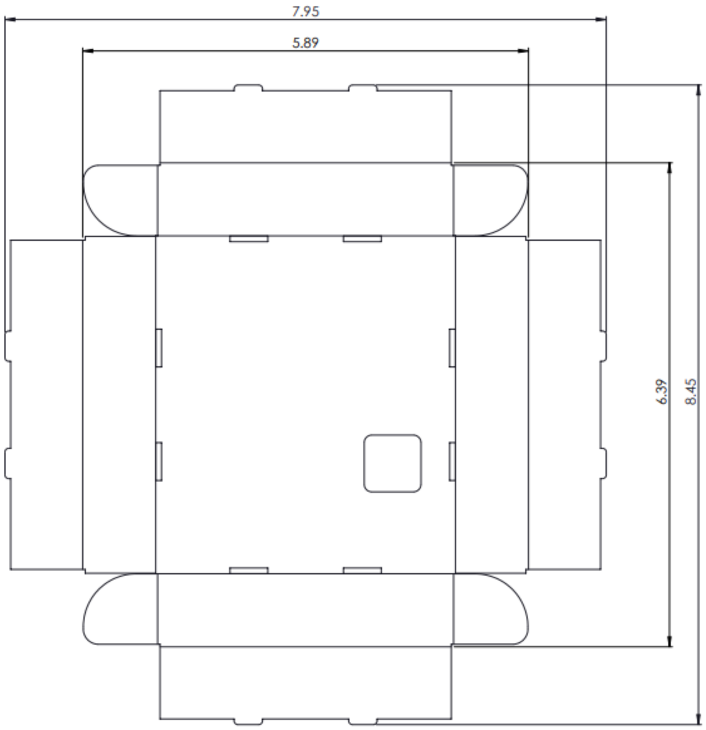

(c) Unfolded with dimensions (inches)

The flat pattern has tabs along each of the four panels that form the sides of the box that insert into slits cut on the main, center panel of the box. These tabs help keep the walls of the box upright and also give the box's walls a double layer of material for added strength. The panels along the width of the box have tabs sticking out to the side that fold under the panels along the length of the box to stabilize the side walls.

Both sheath and box still have a cut-out squares on the back in order to make the on/off power switch accessible to the user. The battery pack sits at the bottom of the box and is attached using Velcro dots.

Test Tube Stand

The design eliminates the need for test tube and lighting racks. Instead, test tube stands were designed to hold the samples. They hold the samples in pre-cut holes and are able to act as test tube stands when outside of the box. The LED strips, which come with an adhesive backing, can easily be stuck onto the top of the battery pack and positioned directly under the test tube stands to provide light to the samples. The test tube stands still have two different versions; one to accommodate 2.0 mL tubes and another to accommodate 0.6 mL tubes. The unfolded and folded views of both stands are shown in Figures 4 and 5.

Figure 4. Small Test Tube Stand (0.6 mL) and Large Test Tube Stand (2.0 mL)

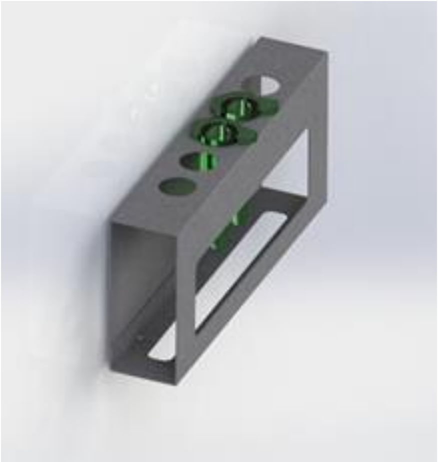

(a) Folded, large

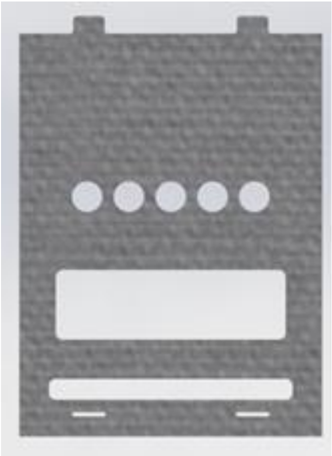

(b) Unfolded, large

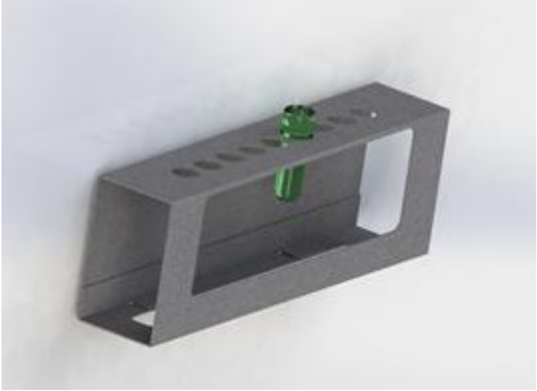

(c) Folded, small

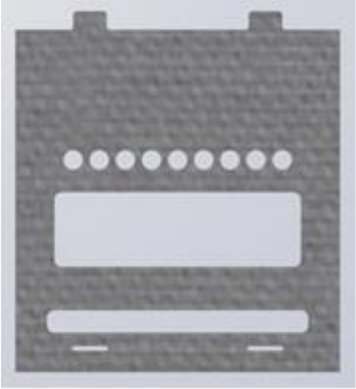

(d) Unfolded, small

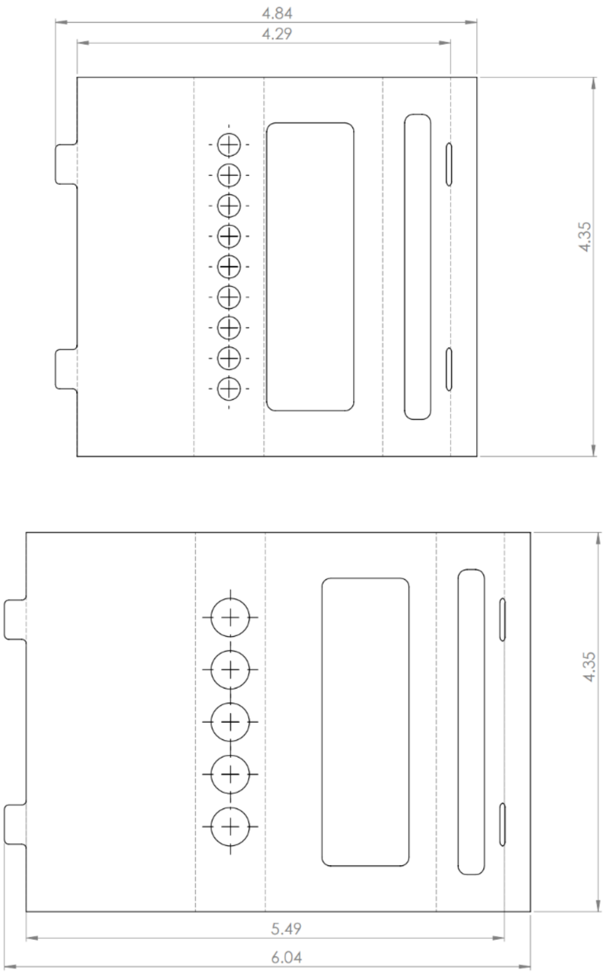

(e) Unfolded small and large test tube stands with dimensions (inches)

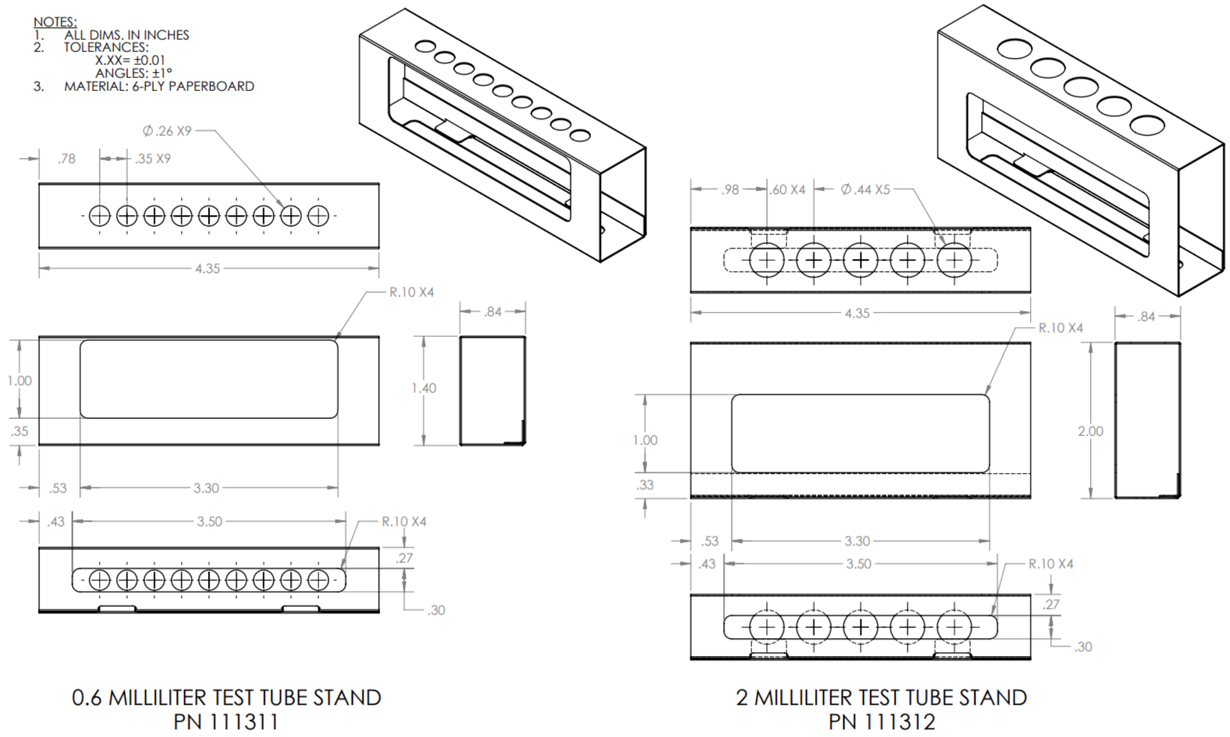

(f) Folded small and large test tube stands with dimensions (inches)

The length and width for both test tube stands are 8 inches and 4.3 inches, respectively. The test tube stand for the 0.6 mL tubes has a height of 1.3 inches and a pattern of 9 holes for the tubes. The test tube stand for the 2 mL tubes has a height of 1.9 inches and a pattern of 5 holes for the tubes.

The stands are folded from their flat pattern into a small box that locks with tabs that insert into slits at the bottom. It also has a viewing window, similar to the sheath. The holes for the test tubes are located on the top face of the box, while the viewing window is located on the front face. Another smaller window is at the bottom of the box, where the LED light can shine through to hit the samples. When the test tubes are placed in the stand, the bottom of the tubes (where the samples are) can be viewed by the user through the viewing window.

Electrical Circuit

Our team determined that the actions of lighting and heating did not need to occur at the same time and be part of the same circuit. A USB heater was utilized so the heating action could be performed independently by plugging the heating element into a computer USB port or wall outlet. Also, the heating element was moved from inside the box to the sheath, on the panel opposite of the filter. Therefore, in order to heat samples, the sheath can be slid with the heater side over the box opening so the heater faces the samples.

The resulting electric circuit for the lighting system consists of the 9V battery and a 3-inch LED strip. A switch on the 9V battery holder cuts off the flow of current from to the LEDs. The heater forms its own independent circuit with the power source that the USB plugs into.

The Cricut

1. Plug in the power cable to the back of the Cricut and press the Power Button to power on the Cricut.

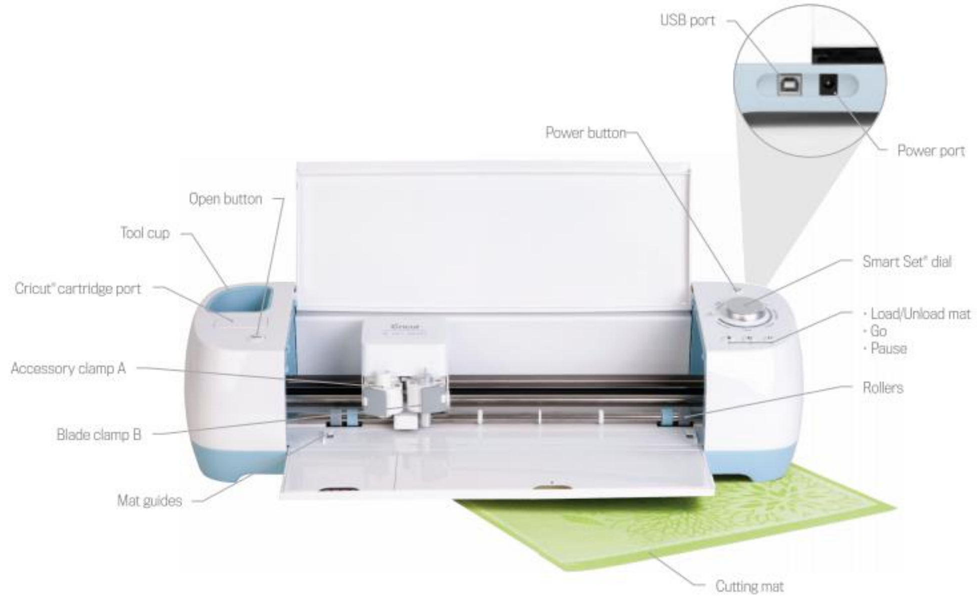

2. On your laptop's browser, copy and paste the following link which will direct you to download and set up the Cricut software: https://design.cricut.com/#/launcher

3. Once you have created an account and set up the Cricut design space on your laptop, copy and paste the following link to your browser which will direct you to the illuminator's Github repository: https://github.com/CPilluminators/Fold-illuminator

4. You'll be taken to the CPilluminator's Fold-illuminator repository where you can download the necessary files to setup your own cutting patterns in Cricut's maker space. Click on "Download Zip" to download the files.

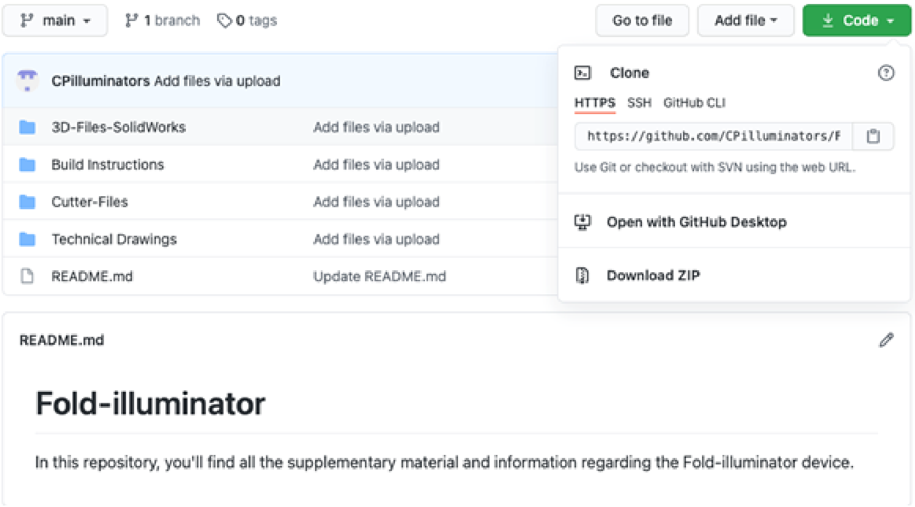

5. Under the “Cutter-Files” file path, you’ll find the necessary “Cut Pattern” and “Fold Pattern” files to use in the Cricut maker space software. Along with those files, you’ll find the associated “Flat Pattern” files that provide the proper scaling dimensions for the cut and fold pattern files.

6. Watch “Kit manufacturing Video” in CPilluminators YouTube channel using the links below on how to setup the cutout using the provided files in the Cricut maker space software.
   Channel Link: https://www.youtube.com/channel/UCoxxTKNHAJlapybWZ8iRnKw
   Video Link: https://youtu.be/3BL6wxpkZuU

7. Once your cutout setup is complete on the maker space, click on “Make it”.

8. The layout on the mat will be shown. The red line on the mat represents the area the Cricut is able to cut/score. The area is 1cm away from all 4 borders of the Cricut mat. Click on “Continue.”

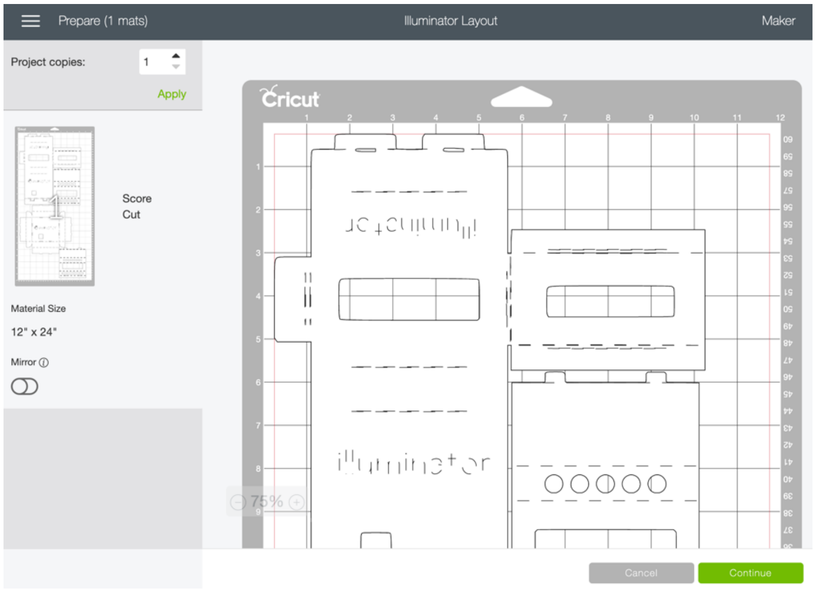

9. Connect to your Cricut Maker through Bluetooth, by clicking the "Connect Machine" button on top. If you don't have a Bluetooth, you may also connect to the Cricut Maker through the provided USB cable. Attach the USB cable to the port at the back of the Cricut maker and also to your laptop.

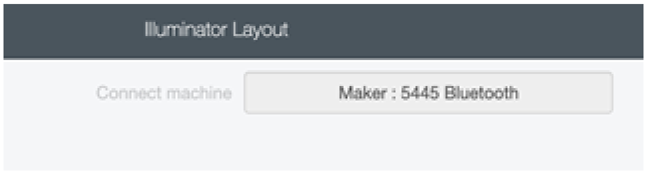

10. Once connected, click on "Browse All Materials" and under category "Cardstock," find and select "Heavy Cardstock – 100lb". "Heavy Cardstock" will show up under select material, click on it again.

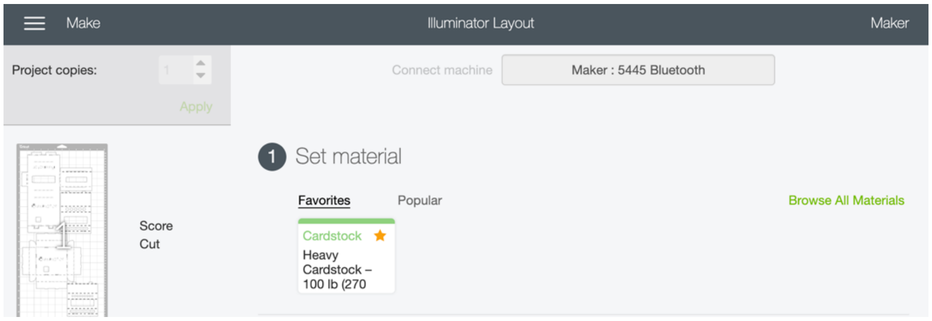

11. A “Pressure” menu drop down will appear. Select the “More” pressure mode.

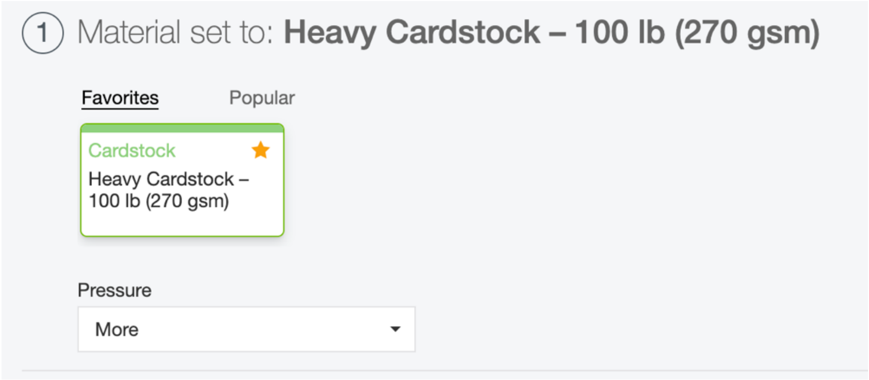

Prepping the Mat

1. Cut the 22”x28” Railboard paper into half along the dashed line with scissors so it is 11”x28”.

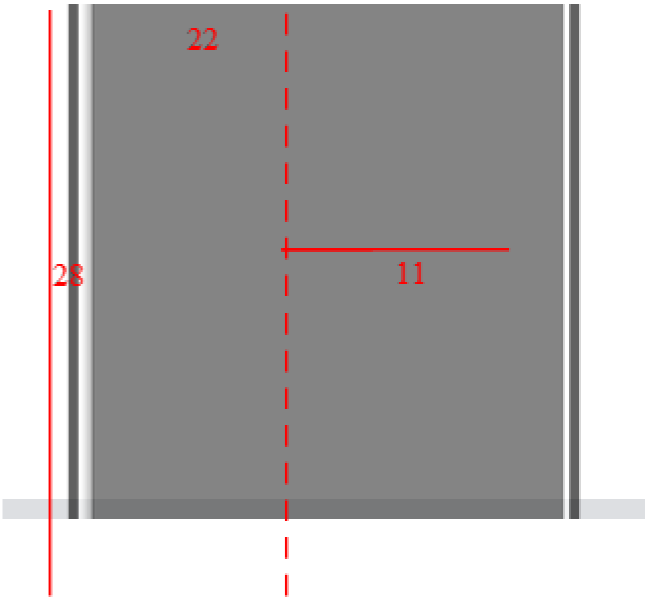

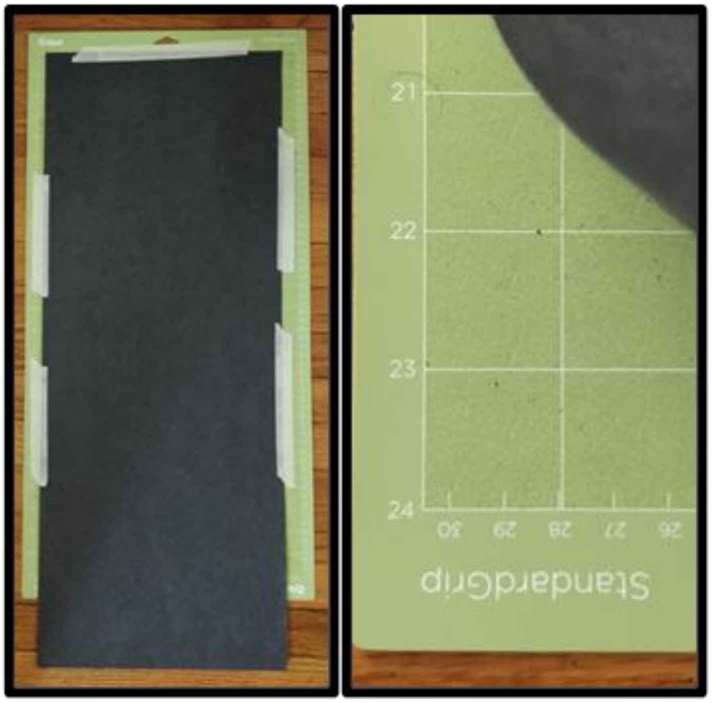

2. Next, lay the paper onto the 24” grip-mat. Make sure to line up the top of the paper to the top horizontal border of the mat.

   Align the left side of the paper 1 centimeter away from the right border of mat. The

easiest way to do so is to align the paper vertically to the “30” cm mark denoted on the bottom of the mat.

If the grip on the mat is not strong, make sure to tape the edges of the paper onto the map so it doesn’t come off during the cutting process.

Why the weird alignment? We do this to be able to cut 2 illuminators from one sheet of paper and also the fact that the Cricut doesn’t cut/use any paper left of the 30 cm mark.

Scoring and Cutting Processes

1. Load the prepared mat onto the Cricut. Make sure to feed the mat under the mat guide notches (circled).

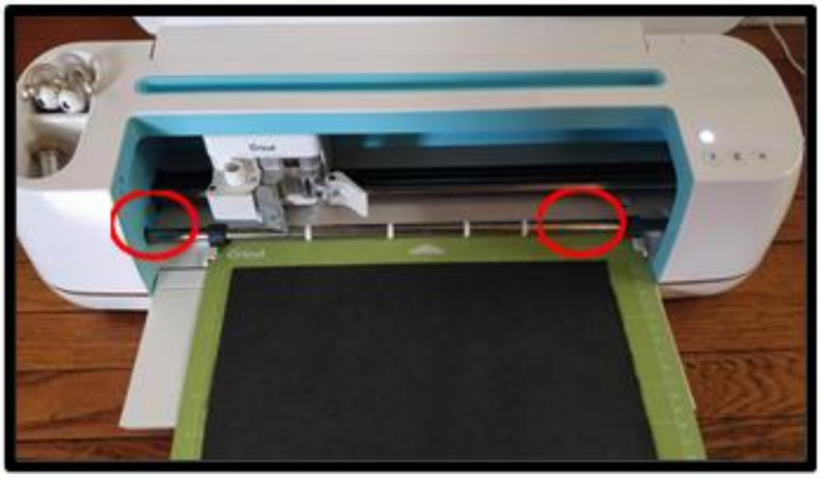

2. Once the mat is in contact with the black rubber wheels behind the notches, press the "Load/Unload" button.

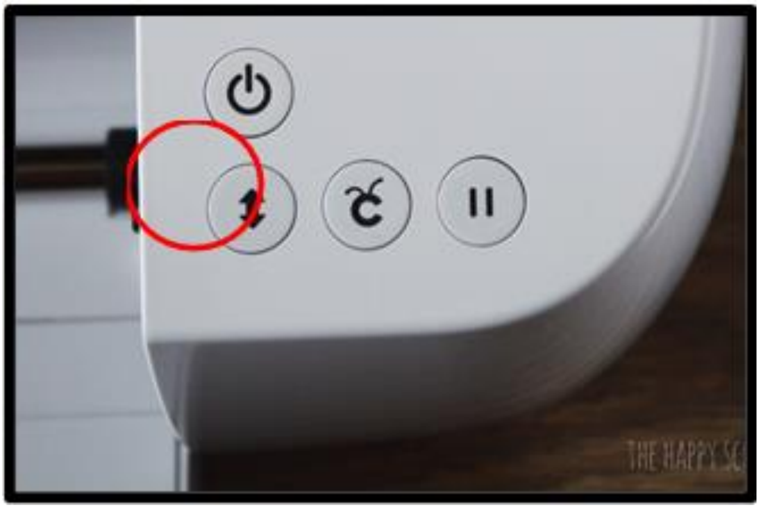

3. Load the scoring wheel onto “Accessory Clamp B” and close the clamp.

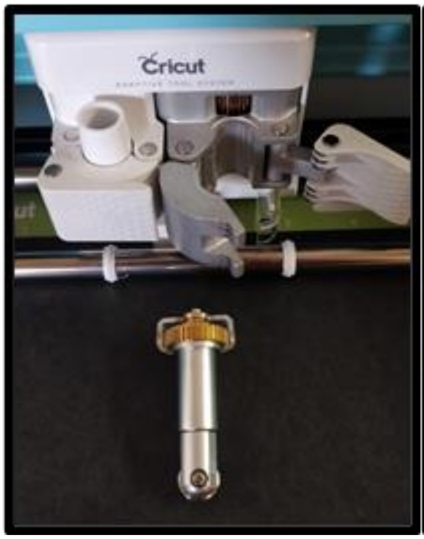

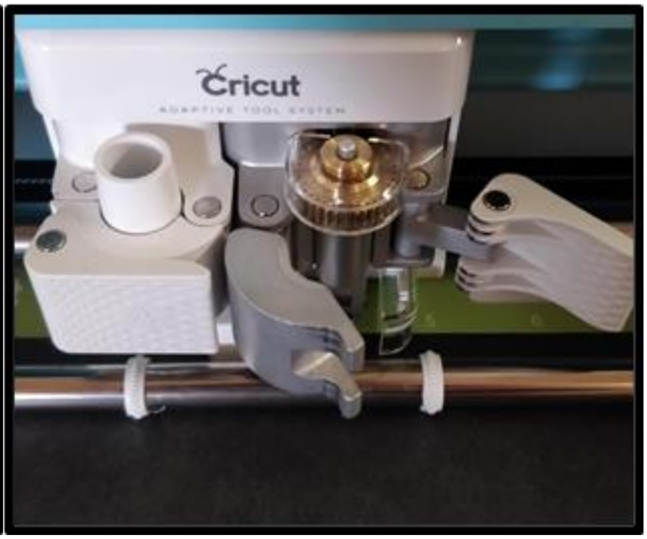

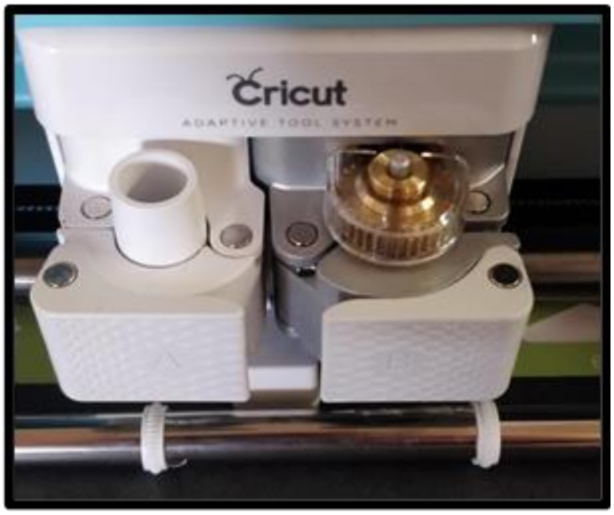

4. Press the “Go” button on the Cricut to begin the scoring process. This should take about 7 minutes.

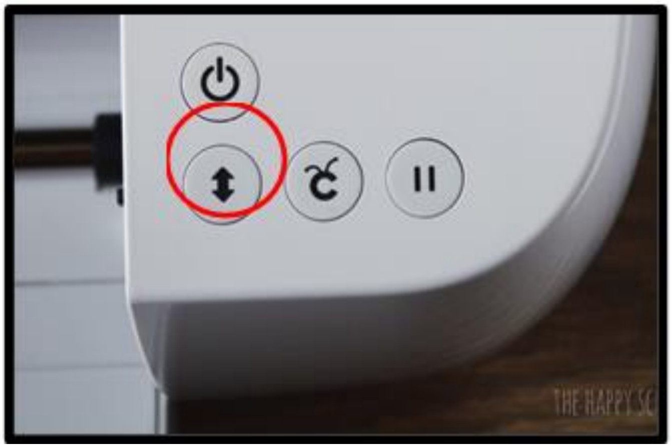

5. Once the scoring process is complete, you’ll be prompted to insert the blade into Clamp B by the Cricut Software. Both the Fine Point Blade (silver), or the Deep Point Blade (black – not pictured) will work for this process. Load the blade onto Clamp B and press the Go button.

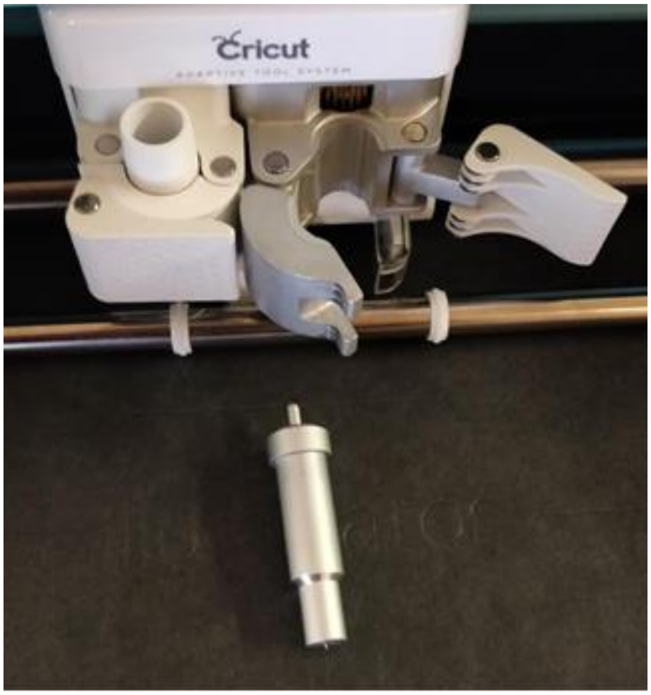

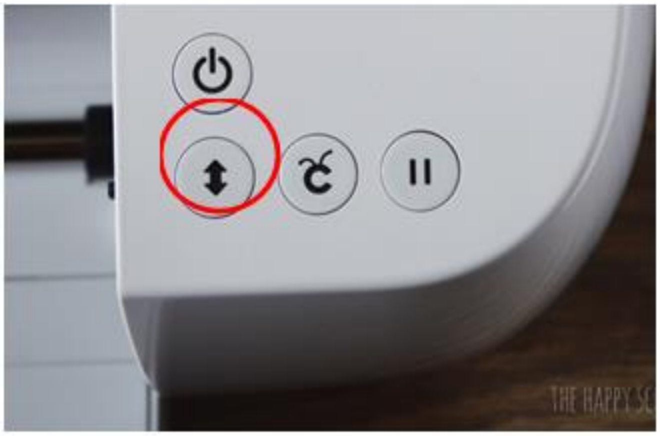
THE HAPPY SC

6. The cutting process will take about (7 minutes). Once it’s complete, unload the mat by pressing the “Load/Unload” button.

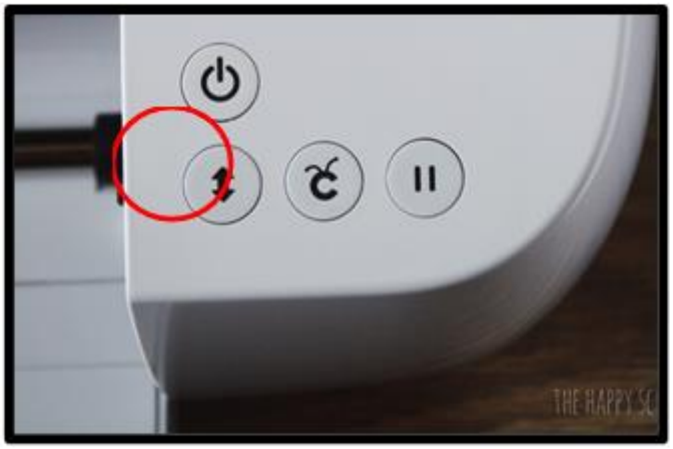

7. This is what the final scored/cut product will look like. Remove/recycle the excess paper and retrieve the cutouts.

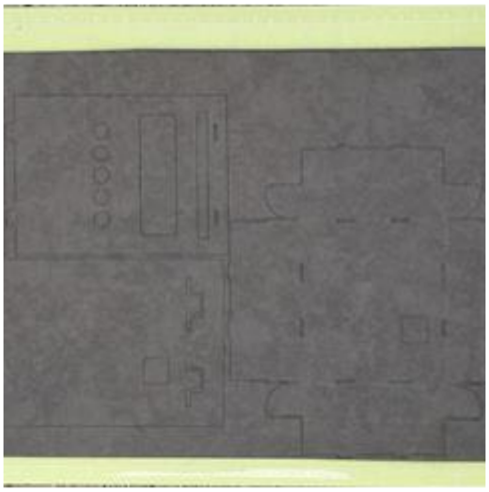

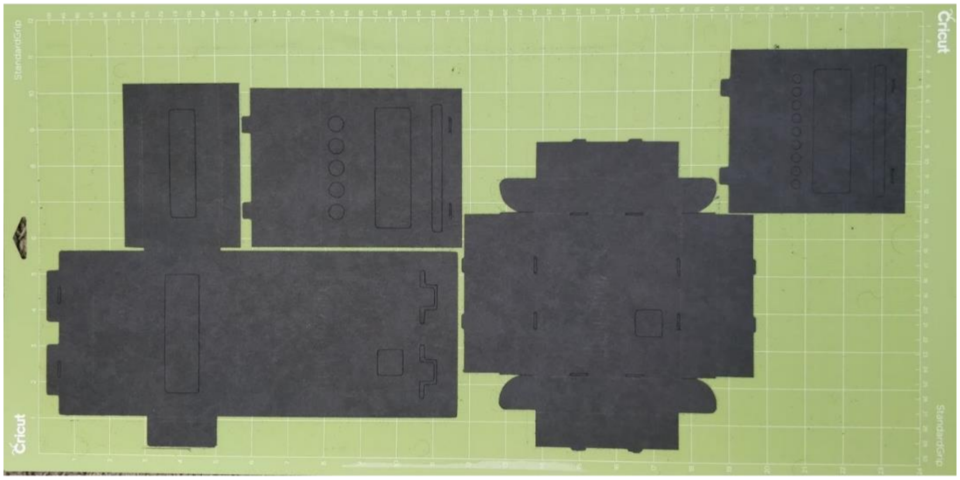

Hint: The easiest way to remove the cutouts from the mat is to bend the mat where the locations of the cutouts. The cutouts themselves will un-attach themselves from the grip-mat.

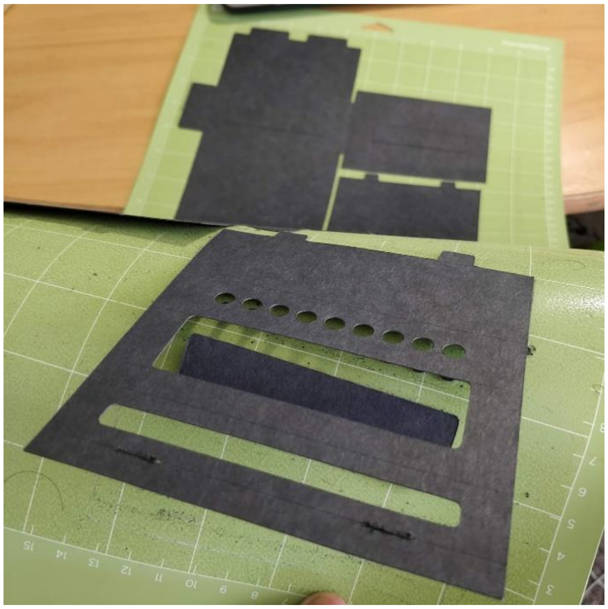

Operation and Safety

WARNINGS:

1. Light can be bright if not covered by filter, do not stare at for long extended periods of time.
2. When not in use, to preserve battery life and protect eyes from unexpected flashes, turn off LED blue lights with battery switch or unplug heater from source.
3. DO NOT operate lights and heater at the same time.

Illumination

1. Choose appropriate test tube stand for samples; either 0.6 mL or 2 mL test tube stand. Place upright and slide tubes into holes. Place test tube stand inside of the box on top of the battery pack, making sure that the cut-out at the bottom of the test tube stand is positioned over the LED light strip.
2. Slide sheath over box with the filter side over the open face of the box, covering it entirely. Make sure that filter is securely attached and positioned over the test tube viewing slot. Also, make sure that the two cut-outs for the battery pack switch on the back of the box and sheath align with one another.
3. Toggle battery pack switch from the back cut-out to turn lights on and off and view fluorescent glow. Turn lights off before removing sheath and taking out samples.

Heating

1. Choose appropriate test tube stand for samples; either 0.6 mL or 2 mL test tube stand. Place upright and slide tubes into holes. Place test tube stand into box over the battery pack.
2. Slide sheath over box with the filter side over the open face of the box, covering it entirely.
3. Take USB cable and plug into wall outlet (with USB-plug converter) or computer USB port to provide power to the heating element and heat the test tubes.
4. Wait until reaction is complete before unplugging USB cable and preparing for illumination viewing.
